# Novel Structural Variation and Evolutionary Characteristics of Chloroplast tRNA in *Gossypium* Plants

**DOI:** 10.3390/genes12060822

**Published:** 2021-05-27

**Authors:** Ting-Ting Zhang, Yang Yang, Xiao-Yu Song, Xin-Yu Gao, Xian-Liang Zhang, Jun-Jie Zhao, Ke-Hai Zhou, Chang-Bao Zhao, Wei Li, Dai-Gang Yang, Xiong-Feng Ma, Zhong-Hu Li

**Affiliations:** 1 Key Laboratory of Resource Biology and Biotechnology in Western China, Ministry of Education, College of Life Sciences, Northwest University, Xi’an 710069, China; zhangtt@stumail.nwu.edu.cn (T.-T.Z.); 202021088@stumail.nwu.edu.cn (Y.Y.); songxiaoyu@stumail.nwu.edu.cn (X.-Y.S.); 202032597@stumail.nwu.edu.cn (X.-Y.G.); 2 State Key Laboratory of Cotton Biology, Institute of Cotton Research, Chinese Academy of Agricultural Sciences, Anyang 455000, China; zhangxianliang@caas.cn (X.-L.Z.); zhaojunjie@caas.cn (J.-J.Z.); zhoukehai@caas.cn (K.-H.Z.); zhaochagnbao@caas.cn (C.-B.Z.); liwei@caas.cn (W.L.); yangdaigang@caas.cn (D.-G.Y.); 3 School of Agricultural Sciences, Zhengzhou University, Zhengzhou 450001, China

**Keywords:** cotton, chloroplast tRNA, evolution, phylogenetic relationship, structural variation

## Abstract

Cotton is one of the most important fiber and oil crops in the world. Chloroplast genomes harbor their own genetic materials and are considered to be highly conserved. Transfer RNAs (tRNAs) act as “bridges” in protein synthesis by carrying amino acids. Currently, the variation and evolutionary characteristics of tRNAs in the cotton chloroplast genome are poorly understood. Here, we analyzed the structural variation and evolution of chloroplast tRNA (cp tRNA) based on eight diploid and two allotetraploid cotton species. We also investigated the nucleotide evolution of chloroplast genomes in cotton species. We found that cp tRNAs in cotton encoded 36 or 37 tRNAs, and 28 or 29 anti-codon types with lengths ranging from 60 to 93 nucleotides. Cotton chloroplast tRNA sequences possessed specific conservation and, in particular, the Ψ-loop contained the conserved U-U-C-X3-U. The cp tRNAs of *Gossypium* L. contained introns, and cp tRNA^Ile^ contained the anti-codon (C-A-U), which was generally the anti-codon of tRNA^Met^. The transition and transversion analyses showed that cp tRNAs in cotton species were iso-acceptor specific and had undergone unequal rates of evolution. The intergenic region was more variable than coding regions, and non-synonymous mutations have been fixed in cotton cp genomes. On the other hand, phylogeny analyses indicated that cp tRNAs of cotton were derived from several inferred ancestors with greater gene duplications. This study provides new insights into the structural variation and evolution of chloroplast tRNAs in cotton plants. Our findings could contribute to understanding the detailed characteristics and evolutionary variation of the tRNA family.

## 1. Introduction

Chloroplasts (cp) are unique oblate organelles equipped with important photosynthesis functions [1]. Besides the complete photosynthetic components, they have also been reported as active organelles participating in other diverse biomolecule processes including biosynthesis of starch, fatty acids, pigments, amino acids, etc. [2,3,4]. Some previous studies found that chloroplasts originated from cyanobacterial ancestors [5,6]. For most plants, the cp genome is a double-stranded circular unit involving four main regions: two inverted repeats (IRs) separated by a large single copy (LSC) region and a small single copy (SSC) region [7,8]. In addition, the features of non-recombination and maternal inheritance (in most angiosperms) of the cp genome provide the opportunity to employ it as material for evolutionary and genomics research [9,10]. There are many genes, such as protein-coding genes and multiple tRNA genes, present in the cp genome [11]. Relevant studies have shown that most of these genes play a role in the organization of photosynthesis and biochemical reactions [12,13,14]. However, it is worth noting that the genetic and mutational characteristics of cp genes, especially cp tRNA genes in angiosperms, still need research.

Generally, single nucleotide polymorphisms (SNPs) and insertions/deletions are the basis of the differences between most alleles. With the characteristics of high abundance, a fairly low mutation rate, and the adaptability to automatic genotyping, SNPs are employed more frequently than other genetic markers, such as microsatellites [15]. Insertions and deletions (indels) are essential sources of polymorphic markers for high-resolution genetic mapping of traits and association studies based on candidate genes or possibly the whole genome [16]. Furthermore, SNPs and indels are also important in the nucleotide sequence evolution analysis of genomes. The calculation of single nucleotide variation may help estimate and comprehend genetic variations of different genome regions.

According to previous studies, the residue polynucleotide sequences of tRNAs fold back, forming clover leaf-like structures with hydrogen bonds, and then turn into L-shaped tertiary structures [17]. The secondary structure of tRNA contains an acceptor arm, dihydrouridine arm (D-arm), dihydrouridine loop (D-loop), anti-codon arm, anti-codon loop, variable loop, pseudouridine arm (Ψ-arm), and pseudouridine loop (Ψ-loop) [18,19]. With this specific structure, tRNA plays an important role in protein synthesis by carrying amino acids to the ribosome [20,21]. Recently, researchers conducted a series of analyses related to tRNAs on the genomic level. Asymmetric combinations and divided segments in tRNA genes would help to understand the diversity of tRNA molecules [22]. Additionally, studies showed that the heterogeneous tRNA fragments play multiple roles in terms of size, nucleotide composition, biogenesis, and even biological disease [23]. Additionally, wobble modifications were frequently found in tRNAs in diverse species after the discovery of tRNA molecules [24,25,26]. In recent years, studies on the genomic structure and evolution of tRNAs in plants has been attracting researchers’ attention. For example, analyses of detailed molecular aspects in cyanobacterial tRNAs, complete genomic features of tRNAs in *Oryza sativa* L, and the evolutionary perspective of chloroplast tRNAs (cp tRNAs) in some economic monocots were previously conducted [27,28,29].

*Gossypium* L., or the cotton plant, is an important economic and oil crop that has been cultivated worldwide. *Gossypium* is a large genus belonging to the angiosperm family Malvaceae. Fryxell divided the cotton genus into four subgenera, with a total of 51 species, including 46 diploid (2*n* = 2× = 26) and five tetraploid (2*n* = 4× = 52) species [30]. The chromosome composition of tetraploid cotton is heterogeneous A and D: *Gossypium hirsutum* L. (AD1), *G. barbadense* L. (AD2), *G. tomentosum* Nuttalex Seemann (AD3), *G. mustelinum* Miersex Watt (AD4), and *G. darwinii* Watt (AD5). A total of eight chromosomes of diploid cotton belong to groups A–G and K. Among them, *G. hirsutum*, *G. barbadense*, *G. arboreum* L., and *G. herbaceum* L. are cultivars, while the others are wild species. Nowadays, the most widely cultivated cotton species are tetraploid *G. hirsutum* and *G. barbadense* [31]. At present, with the rapid development of genome sequencing technology, whole-genome sequences of cotton have been released, which provide the foundation for further analysis of cotton genomes [32,33]. The genome-wide landscape of genomic variation of cotton was constructed through SNP distribution density detection, and functional genes that encode proteins involved in regulation of tissue growth, stress responses, and disease resistance were reported [34,35,36,37,38,39]. Moreover, a previous study on variations of repeat sequences and cp evolutionary relationships detected divergence hotspots in plastid genomes and helped to understand phylogenetic relationships among major *Gossypium* lineages [40]. However, the evolutionary patterns of *Gossypium* chloroplast tRNAs are still unclear. The study of the genomic and evolutionary characteristics of cotton cp tRNAs seems to be significant.

In this study, we investigated ten globally representative cotton cp genomes, including eight diploid and two allotetraploid species. The aims of our study were: (1) identify the genomic characteristics and diversification of cp tRNAs in cotton; (2) analyze the evolutionary relationship of introns in cp tRNA genes; (3) estimate the evolutionary characteristics of SNPs and the indel mutation rate of the cotton chloroplast genome; and (4) investigate the evolutionary pattern of cp tRNAs in cotton species.

## 2. Materials and Methods

### 2.1. Identification of tRNAs

We downloaded the 10 cotton cp genomes (8 diploid and 2 allotetraploid species: *Gossypium arboreum* L., *G. anomalum* Wawra and Peyritsch, *G. robinsonii* (F. Muell.) J. H. Willis, *G. klotzschianum* Andersson, *G. somalense* (Gurke) J. B. Hutch., *G. longicalyx* Hutchinson and Lee, *G. bickii* Prokhanov, *G. populifolium* (Benth.) F. Muell., *G. hirsutum* L., and *G. barbadense* L.) from the National Center of Biotechnology Information (NCBI) (Table 1). These cotton species are widely distributed near the equator and *G. hirsutum*, *G. barbadense*, and *G. klotzschianum* are mainly distributed in the Americas. Subsequently, tRNA gene sequences were identified and extracted from cp genomes without intergenic regions by the GENEIOUS 8.0.2. program [41].

### 2.2. Structural Analysis of tRNAs

ARAGORN and tRNAScan-SE software [42,43] were employed to investigate the secondary structure of tRNA sequences of cp genomes. The default parameters of ARAGORN software were set to investigate tRNAs. The parameters of tRNAScan-SE were set as: bacterial for sequence source, default for search mode, formatted (FASTA) for query sequences, and universal for genetic code for tRNA isotype prediction.

### 2.3. Sequence Alignment

To identify the presence of consensus sequences, sequence alignments were carried out for the intron sequences of the cotton cp tRNAs, *Pinus armandii* Franch., *Marchantia polymorpha* L., *Raphanus sativus* L., *Spirogyra maxima* (Hassall) Wittrock, *Alsophila spinulosa* (Wallich ex Hooker) R. M. Tryon Contr. Gray Herb., *Zea mays* L., *Gleocapsa* sp. PCC 73106, *Nostoc* sp. PCC 7107, and *Nostoc* sp. PCC 7524 using Multalin software, in which default parameters were set [44,45,46].

### 2.4. Phylogenetic Tree Construction

The phylogenetic tree was constructed using MEGA7.0 software [47,48]. To investigate the evolution of chloroplast tRNAs, a matrix of whole tRNA sequences was created by Clustal Omega software before the phylogenetic tree was constructed. MEGA7 software was employed to turn the matrix file into MEGA file format for tree construction, in which the lowest Bayesian information criterion (BIC) was selected for the model. As a result, the Kimura2 + G + I model was found to have the lowest BIC score, 8980.50. Thus, this model was adopted for the phylogenetic tree construction. The other related parameters were as follows: phylogeny reconstruction for analysis, maximum likelihood model, bootstrap method in phylogeny test, 1000 bootstrap replicates, nucleotide type, γ distributed with invariant sites (G + I) model, 5 discrete γ categories, partial deletion for gaps/missing data treatment, 95% site coverage cutoff, and very strong for branch swap filter. 

### 2.5. Analysis of Disparity Index

A disparity index test of pattern heterogeneity was conducted to check the homogeneity of nucleotide substitutions and find whether all substitutions in nucleotides happened at equal rates, i.e., homogeneity in the process of evolution. The statistical parameters were as follows based on a previous study [29]: disparity index test for substitution pattern homogeneity, in sequence pairs, 10,001 Monte Carlo replications, nucleotide for substitution type, partial deletion of gaps/missing data treatment, and site coverage cutoff 95%.

### 2.6. Transition/Transversion Analysis

The transition and transversion rates of the tRNAs genes were analyzed according to their isotypes by MEGA 7 software [49]. The parameters were set as follows: substitution pattern estimation (ML) for analysis, automatic (neighbor-joining tree), maximum likelihood statistical method, nucleotide for substitution type, Kimura 2-parameter model, γ distributed (G) site rates, 5 discrete γ categories, partial deletion of gaps/missing data treatment, 95% of site coverage cutoff, and very strong branch swap filter.

### 2.7. Evolutionary Analysis of Single Nucleotide Polymorphisms

The diversity of single nucleotide polymorphisms of 8 diploid *Gossypium* cp genomes was calculated based on 3 regions: coding regions, introns, and intergenic spacers. The synonymous (dS) and non-synonymous (dN) substitutions of coding genes were also calculated by DnaSP v5.10 software [50]. The coding regions, introns, and intergenic regions of the studied *Gossypium* cp genome were extracted through Geneious 8.0.2 software and aligned manually [41].

### 2.8. Calculation of Mutation Rate

The rate of indel mutation (μ, per site per year) was calculated with the formula:

μ = m/(n*T*), (1)

where m is the number of sites of observed mutation, n is the total number of sites, and *T* is the divergence time of *Gossypium*. The μ value of structural mutations was calculated according to the method of Saitou and Ueda [51]. The *T* value was obtained through relevant published literature searches in the Fossil works database and the Cenozoic Angiosperm Database [52,53]. Additionally, the mutation rates of protein-coding genes and tRNA genes were calculated.

### 2.9. Duplication/Loss Analysis of tRNA Genes

To investigate duplication or loss events of the tRNAs, we used the NCBI taxonomy browser to construct the species tree of *G. arboreum*, *G. anomalum*, *G. robinsonii*, *G. klotzschianum*, *G. somalense*, *G. longicalyx*, *G. bickii*, *G. hirsutum*, *G. barbadense*, and *G. populifolium*. Additionally, the previously constructed phylogenetic tree of the tRNAs was employed as the gene tree. Subsequently, Notung 2.9 software [54,55] was used to reconcile the gene tree and the species tree and obtain the gene duplication and loss nodes.

## 3. Results

### 3.1. Basic Characteristics of Cotton Chloroplast tRNAs

The results of detailed genomic analysis showed that *G. arboreum*, *G. anomalum*, *G. robinsonii*, *G. klotzschianum*, *G. somalense*, *G. hirsutum*, *G. barbadense*, *G. longicalyx*, and *G. populifolium* coded 37 tRNAs, respectively, while only *G. bickii* coded 36 tRNAs (Table 2). The length of the chloroplast tRNAs ranged from 60 nt (tRNA^Gly^ in *G. arboreum*, GCC) to 93 nt (tRNA^Ser^, UGA), with an average length of 76 nt (Table S1).

The genomic analysis results showed that chloroplast tRNA genes of the investigated cotton plants coded 28 or 29 anti-codon types, of which only *G*. *anomalum*, *G*. *longicalyx*, and *G. bickii* coded 29 anti-codons. The most common anti-codons observed in cp tRNAs were UGC-tRNA^Ala^, GCC-tRNA^Gly^, GAC-tRNA^Val^, ACG-tRNA^Arg^, CAA-tRNA^Leu^, GUU-tRNA^Asn^, CAU-tRNA^Ile^, GAU-tRNA^Ile^, and CAU-tRNA^Met^. Additionally, each of these genes, except GCC-tRNA^Gly^ and CAU-tRNA^Met^, had two copies (Table S2). GCC (tRNA^Gly^) was found with one copy in *G. anomalum*, *G. longicalyx*, *and G. bickii* but two copies in the other cotton species. Similarly, CAU (tRNA^Met^) was found with one copy in *G. bickii* but two copies in the other cotton species. There were 33 tRNAs with various iso-acceptors missing in the cp genome of *Gossypium* (Table S2). UCC-tRNA^Gly^ was observed in *G. anomalum*, *G. longicalyx*, *and G. bickii*. All investigated cotton species were shown to possess at least one anti-codon type for each kind of tRNA. Furthermore, the anti-codon CAU was a typical characteristic of tRNA^Met^, which harbored only one type of iso-acceptor. Apart from the existence of the anti-codon CAU in tRNA^Met^, tRNA^Ile^ was also observed to encode the anti-codon CAU in the cotton cp genome (Table S2).

All the tRNA gene families were analyzed by multiple sequence alignment, from which the limited conservation and consensus sequences were found in the Ψ-arm and Ψ-loop. The Ψ-arm of tRNAs was observed to contain the G-G consensus sequence, and the Ψ-loop was observed to contain the conserved sequence U-U-C-X3-U (Table 3).

Most of the tRNAs possessed a G nucleotide at the first position of the acceptor arm, while tRNA^Gln^, tRNA^Pro^, and tRNA^Val^ were observed to contain a U and an A (Table 3). Except for tRNA^Lys^, tRNA^Met^, tRNA^Thr^, tRNA^Tyr^, and tRNA^Val^, other tRNAs contained a G in the first nucleotide of the D-arm. Additionally, there was usually an A at the last position of the D-loop and ANC-loop. At the final site of the D-arm, except for tRNA^Arg^, tRNA^Glu^, tRNA^Ile^, tRNA^Leu^, tRNA^Met^, tRNA^Ser^, and tRNA^Tyr^, the other investigated tRNAs were found to have a C (Table 3). Additionally, we also observed that all tRNA^Lys^, tRNA^Ala^, tRNA^Glu^, tRNA^Arg^, tRNA^Tyr^, and a few tRNA^Leu^ had a C-C-A tail in the 3′ end.

### 3.2. Diversification of tRNA Structure

The tRNA feature of possessing various arms and loops was responsible for protein translation. The results of structure analysis showed that the acceptor arm in cotton chloroplast tRNA contained 6 to 7 nt. Among the 369 investigated tRNA sequences, only nine were found to contain 6 nt, while the remaining 360 tRNAs (97.56%) contained 7 nt (Table S3). In addition, the D-arm was observed to contain 2 to 4 nt, among which 20 contained only 2 nt, and 110 (29.81%) contained 3 nt. The remaining tRNAs (64.50%) were observed to possess 4 nt in the D-arm. Moreover, the D-loop contained 7 to 11 nt. For all the involved tRNAs, 84 contained 7 nt in their D-loops; 69 (18.70%) contained 8; 105 (28.46%) contained 9; 40 (10.84%) contained 10; and the rest (18.98%) contained 11 nt (Table S3).

In all 369 tRNAs, the anti-codon arm had mainly 4 to 5 nt. Among them, 319 (86.45%) had 5 nt, and 50 (13.55%) had 4 nt. In addition, 359 tRNAs (97.29%) contained 7 nt in their anti-codon loops and 10 (only 2.71%) contained 9 nt. This showed that the conservative sequence of anti-codon loops was rather typical (Table 3 and Table S3).

For variable loops, 10 tRNAs (2.71%) contained 2; 30 tRNAs (8.13%) contained 3, 67 tRNAs (18.16%) contained 4; 213 tRNAs (57.72%) contained 5; 39 tRNAs (10.57%) contained 6; and 10 tRNAs (2.71%) contained 8 nt (Table S3). While the Ψ-arms of all the analyzed chloroplast tRNAs were observed to contain 5 nt, most tRNAs (349, 94.58%) had 7 nt in their Ψ-loops, apart from several tRNA^Arg^ (Table S3).

### 3.3. Chloroplast tRNA Contained Introns

**Figure 1:**
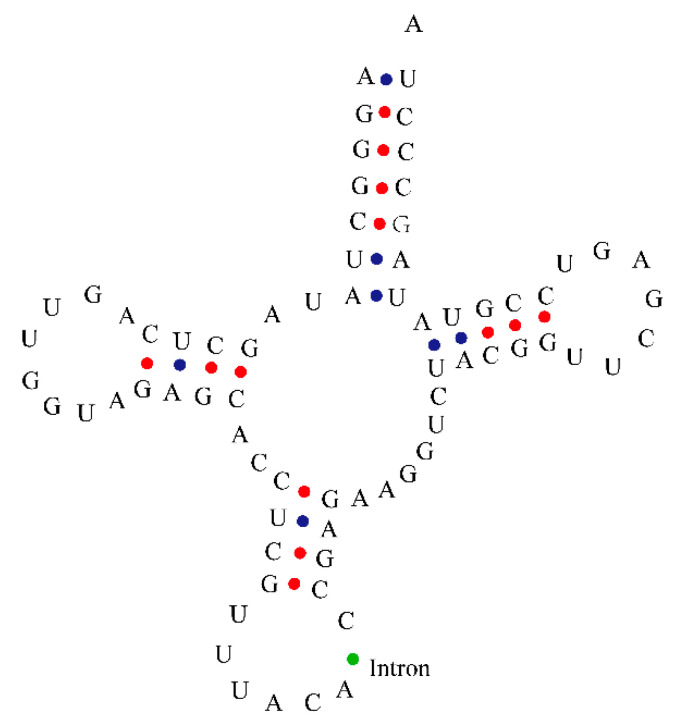
Presence of intron in chloroplast tRNA, found to be located in the tRNA anti-codon loop. The blue dot represents dihydrogen bonds; the red dot represents triple hydrogen bond; and the green dot indicates the position of intron.

**Figure 2:**
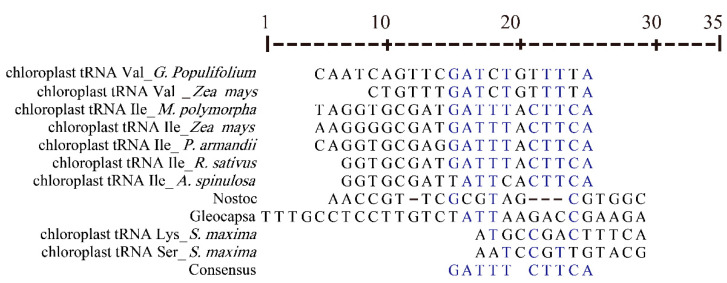
Multiple sequence alignment of introns in tRNA of plant chloroplast and cyanobacteria. Introns in bacterial and chloroplast tRNAs had conserved G-A-T-T-T and C-T-T-C-A consensus sequences.

**Figure 3:**
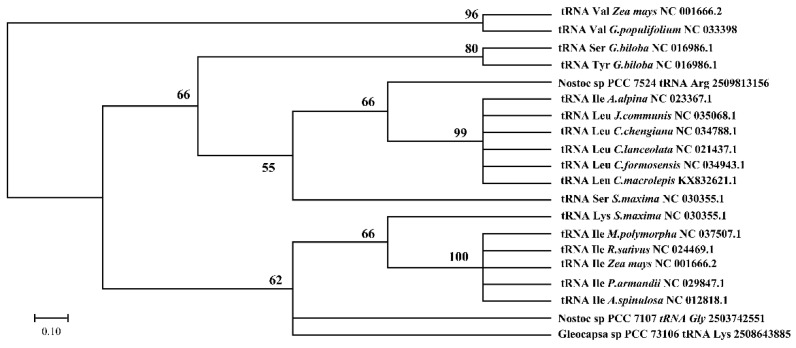
Phylogenetic relationship of introns in chloroplast tRNAs (from angiosperms, gymnosperms, ferns, bryophytes, and algae). Introns of chloroplast tRNA grouped with that of cyanobacteria illustrated a common cyanobacterial origin of introns in chloroplasts. Introns in chloroplast tRNA^Val^ of *Gossypium populifolium* and *Zea mays* indicated the same evolutionary ancestors of corn and cotton introns. Introns present in the same tRNA of plants (tRNA^Ile^ and tRNA^Leu^) tended to appear in the same branch, showing their close phylogenetic relationship.

The cotton chloroplast tRNAs had intron annotations according to previous studies. The tRNA^Val^ of *G. populifolium* was observed to contain an intron in its anti-codon loop region (Figure 1). The introns in bacterial and plant chloroplast tRNAs had conserved G-A-T-T-T and C-T-T-C-A consensus sequences (Figure 2). Phylogenetic analysis showed that chloroplast tRNA introns grouped with the introns in cyanobacteria (Figure 3). Introns contained in the same tRNAs of most plants (tRNA^Ile^ and tRNA^Leu^) tended to appear in the same branch. This revealed their close phylogenetic relationship. The introns in chloroplast tRNA^Val^ of *G. populifolium* and *Zea mays* L. aggregated to the same branch (Figure 3), showing that the introns of corn and cotton have a close phylogenetic relationship.

### 3.4. Chloroplast tRNAs with Non-Typical Features

**Figure 4:**
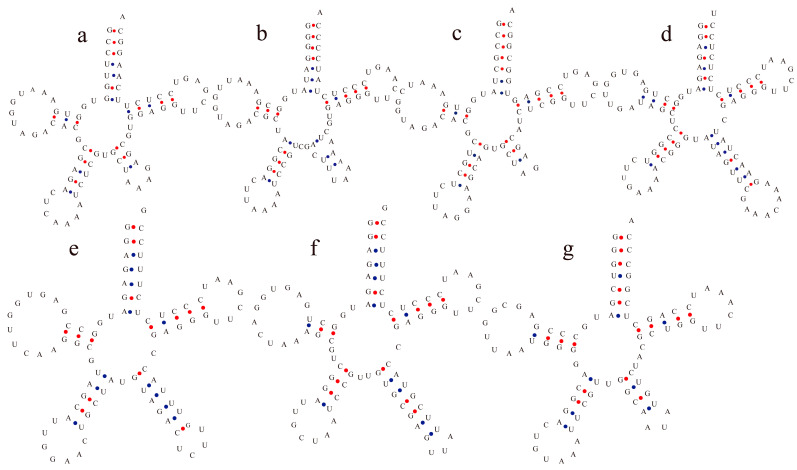
Structures of the chloroplast tRNAs showed the presence of a loop structure in the variable region: (**a**–**c**) tRNA^Leu^; (**d**–**f**) tRNA^Ser^; (**g**) tRNA^Tyr^. Anti-codon loop had seven nucleotides with the conservative X-U-X3-A-A consensus sequence in these tRNAs. Loop structure of the variable region was observed to contain three to eight nucleotides from tRNA^Leu^, tRNA^Ser^, and tRNA^Tyr^.

A few unconventional tRNAs were observed in cotton cp genomes. tRNA^Leu^, tRNA^Ser^, and tRNA^Tyr^ were observed to have a loop in the variable region (Figure 4). In these non-typical tRNAs, the anti-codon loop harbored 7 nt with the X-U-X3-A-A consensus sequence, and the stem of the anti-codon loop contained 4 to 5 nt. The variable loop region was observed to have 2 to 8 nt for tRNA^Leu^, tRNA^Ser^, and tRNA^Tyr^. The stem of the variable region contained 3 to 7 nt pairs. Obviously, tRNA^Ser^ had mainly 6 or 7 nt pairs (Figure 4). These loop structures in variable regions of tRNAs put forward the question of whether these loops play an important role in the process of protein translation of the chloroplast.

### 3.5. Cotton Chloroplast tRNAs Were Derived from Several Evolutionary Ancestors

**Figure 5:**
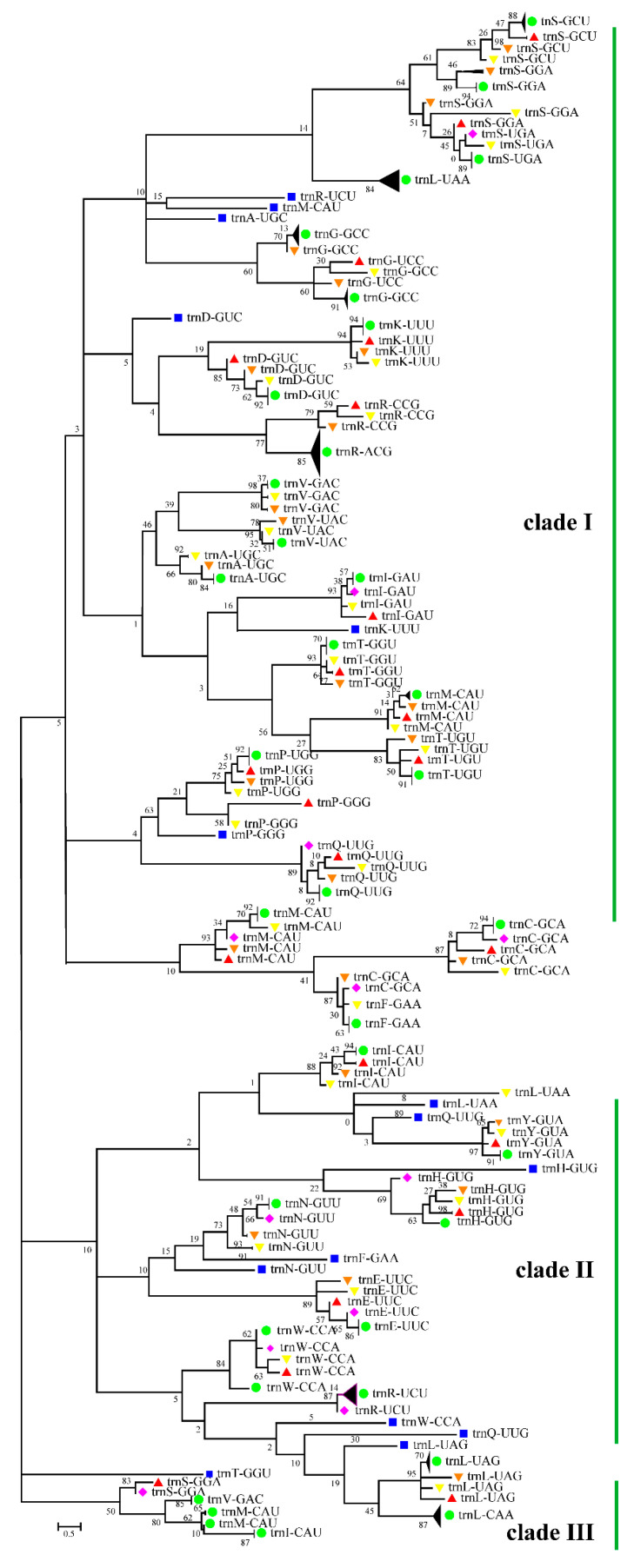
Phylogenetic tree of chloroplast tRNAs. A, alanine (Ala); R, arginine (Arg); N, asparagine (Asn); D, aspartate (Asp); C, cysteine (Cys); Q, glutamine (Gln); E, glutamate (Glu); G, glycine (Gly); H, histidine (His); I, isoleucine (Ile); L, leucine (Leu); K, lysine (Lys); M, methionine (Met); F, phenylalanine (Phe); P, proline (Pro); S, serine (Ser); T, threonine (Thr); W, tryptophan (Trp); Y, tyrosine (Tyr); V, valine (Val). The green solid circle represents *Gossypium*; the red solid triangle represents *P. taeda*; the pink solid diamond represents *A. lyrate*; the yellow solid triangle represents *P. nudum*; the orange solid triangle represents *D. hirsuta*; and the blue solid square represents Nostoc sp. PCC 7524. Phylogenetic analysis showed the polyphyletic origin of chloroplast tRNAs.

The phylogenetic tree of cotton and other various species’ tRNA sequences from a wide range of taxonomic positions, including algae (Nostoc sp. PCC 7524), bryophytes (*Dumortiera hirsuta*), ferns (*Psilotum nudum*), gymnosperms (*Pinus taeda*), and angiosperms (*Arabidopsis lyrata*), presented three major clusters (Table 4). In all, there were 87 groups in integrated clade I, 43 groups in integrated clade II, and 7 groups in integrated clade III. In the phylogenetic tree, tRNA^Ser^, tRNA^Leu^, tRNA^Arg^, tRNA^Val^, tRNA^Ile^, tRNA^Met^, and tRNA^Gln^ were polyphyletic, positioned in more than two integrated clades. In integrated clade I, most of tRNA^Met^ was clustered into two polyphyletic sub-clades (containing nine groups), one embedded in the tRNA^Thr^ group and another adjacent to tRNA^Cys^ and tRNA^Phe^, while tRNA^Met^ of cyanobacteria was independently clustered to its tRNA^Arg^. In integrated clade II, tRNA^Leu^ was closely related to cyanobacterial tRNA^Gln^, even with its polyphyly. tRNA^Trp^, except the cyanobacterial one, was clustered into a monophyletic group, while the cyanobacterial one was embedded into the sister clade of the tRNA^Trp^ group. tRNA^Ile^ was found in all three clades. Interestingly, tRNA^Leu^, tRNA^Ile^, tRNA^Gln^, tRNA^Phe^, and tRNA^Arg^ present in integrated clade I were also found in clade II, and the tRNAs in integrated clade III were also found in clade I (Figure 5).

### 3.6. Transition/Transversion of tRNAs

**Figure 6:**
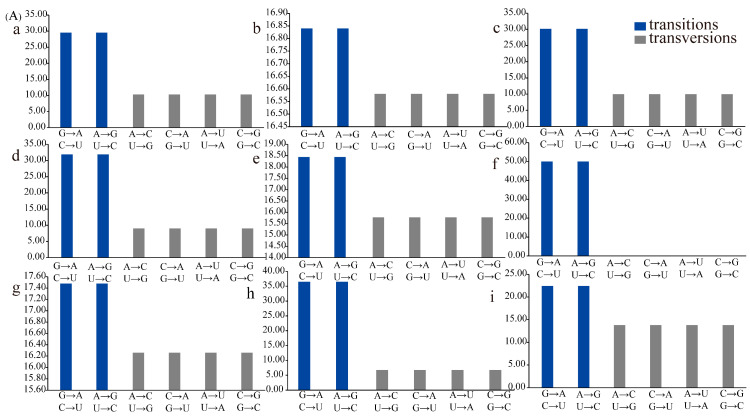

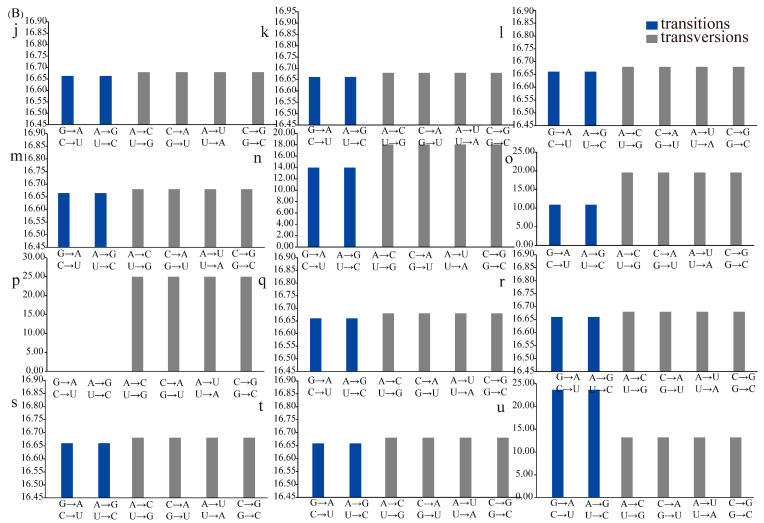
Rates of transition (blue) and transversion (gray) of chloroplast tRNAs. (**A**) a–i refer to R, Arg; S, Ser; C, Cys; E, Glu; G, Gly; W, Trp; L, Leu; K, Lys; M, Met. (**B**) j–u refer to A, Ala; P, Pro; N Asn; D, Asp; T, Thr; Q, Gln; H, His; I, Ile; Y, Tyr; V, Val; F, Phe; overall.

tRNAs have evolved with almost equal transition and transversion rates in spite of the small probability of transition or transversion events in tRNAs. In the present study, we found some intriguing phenomena in the substitution rate of cotton chloroplast tRNAs. A transition rate of 16.66 and transversion rate of 16.68 were found in tRNA^Ala^, tRNA^Phe^, tRNA^Asp^, tRNA^Pro^, tRNA^Tyr^, tRNA^Val^, and tRNA^Ile^. This showed that the transversion rate was slightly higher than the transition rate, and these tRNAs evolved with almost the same transition and transversion rates (Figure 6, Table S4). The highest transition rate was 50.00 for tRNA^Trp^ and the highest transversion rate was 25.00 for tRNA^His^. Correspondingly, the lowest transition rate (0.00) was observed for tRNA^His^, and the lowest transversion rate (0.00) for tRNA^Trp^ (Figure 6). This indicated that the tRNA^Trp^ of the cotton cp genome had experienced a high transition rate without any transversion. Similarly, tRNA^His^ had experienced a high rate of transversion but no transition. For tRNA^Lys^, tRNA^Arg^, tRNA^Met^, tRNA^Asn^, tRNA^Cys^, tRNA^Ser^, tRNA^Glu^, tRNA^Gly^, and tRNA^Leu^, the transition rate was higher than the transversion rate (Figure 6A). Additionally, the transition rate was two times higher than transversion for tRNA^Arg^, tRNA^Cys^, and tRNA^Glu^, which showed that the evolution of chloroplast tRNA^Arg^, tRNA^Cys^, and tRNA^Glu^ tended toward transition rather than transversion. For tRNA^Gln^ and tRNA^Thr^, the transversion rate was apparently higher than the transition rate (Figure 6B). This indicated that these tRNA iso-acceptors experienced transversion substitutions more easily than transition. The substitution rates of overall cp tRNAs showed that the average transition rate (23.64) was greater than the transversion rate (13.18) (Figure 6B). 

### 3.7. Evolutionary Characteristics of Single Nucleotide Polymorphisms

The biallelic and parallel mutation SNPs of the eight diploid *Gossypium* cp genomes were calculated. There were 2709 SNPs in *Gossypium* cp genomes (Table 5). They were subdivided into coding, intron, and intergenic spacer regions for further analyses. Among the 2709 SNPs, 906 were in coding regions, 299 were in intron regions, and 1504 were in intergenic spacers. The percentage of SNPs to total length was 1.14, 1.39, and 2.90%, respectively. In coding regions, the overall ratio of nonsynonymous mutations to synonymous mutations (dN/dS) was 3.04.

### 3.8. Mutation Rate of Chloroplast Genome

The divergence time of *Gossypium* was speculated to be 9.8 Mya from *Theobroma* [52,53]. The total length of the eight diploid cotton cp genomes was 156,796 bp. The mutation rate (μ_1_) of the protein-coding genes and the mutation rate (μ_2_) of the tRNA genes were calculated using the length of genomes, the number of indel mutations (522 for protein-coding genes and 133 for tRNA genes), and the divergent time. The value of μ_1_ of 0.34 × 10^–9^ per site per year for protein-coding genes and the value of μ_2_ of 0.09 × 10^–9^ per site per year for tRNA genes were obtained, respectively.

### 3.9. tRNA Duplication/Loss Events

Besides substitution, duplication and loss events of genes had a vital influence on their evolution. The analysis of duplication or loss events indicated that the investigated cotton chloroplast tRNA genes had experienced 226 duplication and 93 co-duplication events (Figure S1). However, only 63 loss events were observed (Figure S1, Table S5). The results showed that the duplication of genes was greater than the loss of genes for all of the tRNAs.

## 4. Discussion

The nucleotide composition of tRNA is closely related to its senior structure, which is responsible for the translation process. In many species, the tRNA family is conserved in evolution [56,57]. As one of the major gene components of semi-autonomous chloroplast, tRNAs were shown to have several basic conserved genomic features. tRNA^Leu^ and tRNA^Ser^ were observed to have 80 nt or more. These two tRNA isotypes were also found to harbor more than 83 nt in Adoxaceae plants, which shows that the gene sequences of tRNA^Ser^ and tRNA^Leu^ are longer than other tRNAs [58]. In addition, some tRNA iso-acceptors were not observed in the cp genome of *Gossypium*, which was similar to that of the species in Gramineae [28]. Additionally, this is perhaps related to the codon usage and wobble base pairing in the genomic constitution of plants [59]. Furthermore, selenocysteine and suppressor tRNAs were found lacking in the cotton cp genome, which were present in *Oryza sativa*, *Sachharum officinarum* L., *Sorghum bicolor* (L.) Moench, *Triticum aestivum* L., and *Zea mays*. This may be related to the biotoxin accumulation ability of these two tRNAs [29]. The differences between the quantity (36 or 37) and anti-codon types (28 or 29) of tRNAs in diploid and allotetraploid cotton species were not significant. This may be related to the stability and conservation of tRNA genes and the long cultivation of cotton species, which reduced the level of interspecific diversity [60,61].

The tRNA family was conserved in its genomic composition. In this study, most of the involved tRNAs were observed to harbor the conservative sequence U-U-C-X3-U at their Ψ-loop (Table 3). Additionally, in Adoxaceae, U-U-C-A conservative nucleotides at its Ψ-loop region were found [58]. This showed that the short consensus sequences might be important components of the molecular recognizer in the Ψ-loop, and are associated with the recognition of ribosomal RNA during the translation process [62]. In our study, we found that the acceptor arm of cotton chloroplast tRNA contained 6 to 7 nt; the D-arm contained 2 to 4 nt; the D-loop contained 7 to 11 nt; the anti-codon loops contained 7 and 9 nt; the Ψ-arms contained 5 nt; and the Ψ-loops had 7 nt. Additionally, in gymnosperm chloroplast genomes, the acceptor arm of tRNAs harbors 6 bp to 7 bp; the D-arm has 3 bp or 4 bp; and the D-loop contains 7 nt to 11 nt; the anti-codon loop contains 7 nt; the Ψ-arm contains 5 bp; and the Ψ-loop has 7 nt [63]. Our results and previous findings together suggest that chloroplast RNAs are significantly conserved, though a few tRNAs contain rare secondary structures [64,65]. In the present study, some tRNA^Leu^, tRNA^Ser^, and tRNA^Tyr^ were found in the variable region including a loop structure. The presence of this unconventional structure in the variable region exhibited the structural variation existing in tRNAs and this might be relevant to the maintenance of tRNA structures and the interaction with the D-arm and Ψ-arm [66].

Introns are previously reported in archaeal and eukaryotic genomes that break the continuity of numerous eukaryotic genes [67]. Here, the intron was observed in chloroplast tRNAs of *G. populifolium*. This is consistent with previous reports in other organisms [68,69]. In general, the group I intron has a few hundred nucleotides because of its built-in ribozyme unit [70]. tRNAs contain sequences of less than 100 polynucleotides that fold into a clover-type secondary structure [71]. Thus, the intron we observed in this study might be a part of intron I that was contained within the cotton chloroplast genome.

Phylogenetic analysis revealed that the introns in chloroplast tRNA^Val^ of *Gossypium populifolium* and *Zea mays* aggregated to the same branch (Figure 3), suggesting that the introns of corn and cotton have a close phylogenetic relationship. Additionally, chloroplast tRNAs with introns were grouped with cyanobacteria, providing supportive evidence for a common cyanobacterial lineage source of cp tRNAs [58].

In addition to the anti-codon CAU of tRNA^Met^, tRNA^Ile^ was observed to code the anti-codon CAU in the cotton chloroplast genome. This may be associated with the modification of tRNA [72]. Three types of tRNAs—tRNA^fMet^, tRNA^Met^, and tRNA^kIle^, with anti-codon CAU—were found in a bacterial genome, and tRNA^kIle^ was able to identify the codon of isoleucine instead of methionine after anti-codon modification [73]. For the tRNA^Ile^ CAU observed in the genomes of plants, the first position in the anti-codon of these tRNAs has a lysidine-like nucleotide. On the other hand, a methionine-discerning anti-codon CAT was found in the genes. After the modification of the C residue of the CAT anti-codon, the mature tRNA shows isoleucine-identifying activity rather than methionine-identifying activity [74,75].

The presence of three obvious clusters and diverse groupings, and the appearance of tRNA groups from different plants with a wide range of taxonomic positions in integrated clade I and II, clade I and III, and clade I, II, and III of the phylogenetic tree, indicate frequent duplication and divergence during their evolution. In addition, tRNA^Ser^, tRNA^Leu^, tRNA^Arg^, tRNA^Val^, tRNA^Ile^, tRNA^Met^, and tRNA^Gln^ were found in more than two integrated clades. This indicates their multiple evolutionary origins. Diverse groupings and the overlapping of tRNA groups from different plants suggest that the tRNAs have several inferred ancestors, including tRNA^Met^, tRNA^Ile^, etc., in the evolutionary history [63]. The interlaced emergence of tRNA groups (tRNA^Met^, tRNA^Thr^, tRNA^Arg^, tRNA^Trp^, tRNA^Val^, and tRNA^Ser^) also suggests that evolutionary relationships among these tRNA types are relatively close.

Just as the cases with the most genetic variations, the transition rate was greater than the transversion rate of *Gossypium* cp tRNAs at the overall level. However, tRNA^Trp^ and tRNA^His^ showed different substitutive choices, which may be caused by various factors, such as their neighbor bases and the efficiency of the repair system of DNA strands [76]. The percentage of SNPs in coding regions, intron regions, and intergenic spacers compared to the total cotton cp genomes was 1.14, 1.39, and 2.90%, respectively, which implies that the density of SNPs was different in the cotton cp genome and the intergenic spacer of *Gossypium* was more variable than intron regions. This may be associated with the richness of A/T and G/C repetitive units in certain regions of the cp genome. It also implies that the evolutionary mutation potential of different positions in the genome is unbalanced [77]. In coding regions, the overall ratio of non-synonymous to synonymous mutations (dN/dS) was 3.04, showing that non-synonymous mutations had been fixed in the cotton cp genome. In the involved diploid *Gossypium* cp genomes, the mutation rate (μ_1_), 0.34 × 10^–9^ per site per year of protein-coding genes, was significantly higher than the mutation rate (μ_2_), 0.09 × 10^–9^ per site per year of tRNA genes (more than three times than that of μ_2_), which also implies that compared with protein-coding genes, tRNA genes and structures have considerable evolutionary stability [78]. In addition, it should be mentioned that the genomic data employed in our study still have limitations. Perhaps the addition of more diverse gene sequences would provide more interesting results.

In concert with multiple factors of tRNA evolution, most important gene functions have evolved from gene duplication events [79,80]. In this study, there were about five times as many duplication events as loss events. Many studies have also shown that frequent duplication greatly promotes evolution and functional diversification in gene families [81,82]. This may provide further insights to confirm that the evolution of cotton tRNAs derived from polyphyletic evolutionary ancestors.

## 5. Conclusions

Cotton chloroplast tRNAs encode 28 or 29 anti-codon types, and 36 or 37 anti-codon-specific tRNAs with lengths ranging from 60 (tRNA^Gly^) to 93 nucleotides (tRNA^Ser^). Thirty-three anti-codon types including AGC-tRNA^Ala^ are absent in the cp genome of *Gossypium*. The CAU anti-codon is encoded in both tRNA^Met^ and tRNA^Ile^. The acceptor arm of cotton chloroplast tRNA contains 6 to 7 nt; the D-arm contains 2 to 4 nt; the D-loop contains 7 to 11 nt; the anti-codon loop contains 7 and 9 nt; the Ψ-arms contains 5 nt; and the Ψ-loop has 7 nt. The Ψ-arm of cotton chloroplast tRNAs contains the G-G consensus sequence, and the Ψ-loop contains the conserved U-U-C-X3-U motifs. Additionally, cotton chloroplast tRNAs were found to contain introns and a few tRNA^Leu^, tRNA^Ser^, and tRNA^Tyr^ were observed to have a loop in the variable region. Furthermore, phylogenetic analysis suggests that tRNAs possibly have several inferred ancestors, including tRNA^Met^, tRNA^Ile^, etc., in the evolutionary history. On the other hand, the average transition rate of all involved cp tRNAs was greater than their transversion rate. The density of SNPs was unbalanced and the intergenic spacer of *Gossypium* was more variable than intron regions. Gene duplication events (226 duplication and 93 co-duplication) have occurred more frequently than gene loss events (63) in cotton chloroplast tRNAs. These results provide helpful insights into the detailed characteristics and evolutionary variation of the tRNA family.

## Figures and Tables

**Table 1:** *Gossypium* species information in the study.

Karyotype	Species	Accession Number	Wild/Cultivars
A2	*Gossypium arboreum*	NC_016712	cultivars
B1	*G. anomalum*	NC_023213	wild
C2	*G. robinsonii*	NC_018113	wild
D3-k	*G. klotzschianum*	NC_033394	wild
E2	*G. somalense*	NC_018110	wild
F1	*G. longicalyx*	NC_023216	wild
G1	*G. bickii*	NC_023214	wild
K2	*G. populifolium*	NC_033398	wild
AD1	*G. hirsutum*	HQ901196	cultivars
AD2	*G. barbadense*	HQ901199	cultivars

**Table 2:** Distribution of tRNA isotypes in cotton chloroplast genome.

tRNA Isotype	Number of tRNAs									
	A2 ^1^	B1 ^2^	C2 ^3^	D3-K ^4^	E2 ^5^	F1 ^6^	AD1 ^7^	AD2 ^8^	G1 ^9^	K2 ^10^
Alanine	2	2	2	2	2	2	2	2	2	2
Glycine	2	2	2	2	2	2	2	2	2	2
Proline	1	1	1	1	1	1	1	1	1	1
Threonine	2	2	2	2	2	2	2	2	2	2
Valine	3	3	3	3	3	3	3	3	3	3
Serine	3	3	3	3	3	3	3	3	3	3
Arginine	3	3	3	3	3	3	3	3	3	3
Leucine	4	4	4	4	4	4	4	4	4	4
Phenylalanine	1	1	1	1	1	1	1	1	1	1
Asparagine	2	2	2	2	2	2	2	2	2	2
Lysine	1	1	1	1	1	1	1	1	1	1
Aspartate	1	1	1	1	1	1	1	1	1	1
Glutamate	1	1	1	1	1	1	1	1	1	1
Histidine	1	1	1	1	1	1	1	1	1	1
Glutamine	1	1	1	1	1	1	1	1	1	1
Isoleucine	4	4	4	4	4	4	4	4	4	4
Methionine	2	2	2	2	2	2	2	2	1	2
Tyrosine	1	1	1	1	1	1	1	1	1	1
Cysteine	1	1	1	1	1	1	1	1	1	1
Tryptophan	1	1	1	1	1	1	1	1	1	1
Selenocysteine	0	0	0	0	0	0	0	0	0	0
Suppressor	0	0	0	0	0	0	0	0	0	0
Total	37	37	37	37	37	37	37	37	36	37

^1^*Gossypium arboretum*; ^2^*G. anomalum*; ^3^*G. robinsonii*; ^4^*G. klotzschianum*; ^5^*G. somalense*; ^6^*G. longicalyx*; ^7^*G. hirsutum*; ^8^*G. barbadense*; ^9^*G. bickii*; ^10^*G. populifolium*.

**Table 3:** Sequence alignment and the presence of isotype-specific conserved nucleotide consensus sequences in the cotton chloroplast tRNAs.

tRNA Isotype	AC-Arm	D-Arm	D-Loop	ANC-Arm	ANC-Loop	Variable Region	Ψ-Arm	Ψ-Loop
Alanine	GGGGAUA	GCUC	AGUUGGUA	CCGCU	CUUGCAU	AUGUC	AGCGG	UUCGAGU
Arginine	GXGXCX_2_	Gx_3_	AX_2_GGAUA	*****	CUXCXAA	GU	GG	UUCGAAU
Asparagine	GUCGGGA	GCUC	AGUUGGUA	GUCGG	CUGUUAA	UGGUC	GUAGG	UUCGAAU
Aspartate	GGGAUUG	GUUC	AAUUGGUCA	CCGCC	CUGUCAA	AAGCU	GCGGG	UUCGAGC
Cysteine	GGCGACA	GCC	GAGCGGUAA	GGGGA	CUGCAAA	UAUUC	CCCAG	UUCAAAU
Glutamate	GX_2_CX_3_	GX_3_	AGXGGUX_1–3_	CX_2_CX	CUUUCAX	X_2_GX_1–2_	X_3_GX	UUCXAXU
Glutamine	UGGGGCG	GCC	AAGUGGUAA	CGGG	UUUUGGU	CUAUGC	GGAGG	UUCGAAU
Glycine	GCGGAUA	GU	CGAAUGGUAAA	UCUCU	UUGCCAA	AGAC	GCGGG	UUCGAUU
Histidine	GCGGAUG	GCC	AAGUGGAUCAA	GUGGA	UUGUGAA	CAUGC	GCGGG	UUCAAUU
Isoleucine	GCAUCCA	GCU	GAAUGGUUAA	CCCAA	CUCAUAA	AAUUC	GUAGG	UUCAAUU
Leucine	GX_6_	GXG	AAAUXGX_3–4_A	X_3_GX	CUX_4_A	XGX_9–12_	X_3_GG	UUCXAGU
Lysine	GGGUUGC	ACUC	AACGGUA	UCGG	CUUUUAA	CUAGUU	CCGGG	UUCGAGU
Methionine	XCX_5–6_	X_3_G	AGUX_5–6_	*****	XUCAUAX	X_2_GUC	AUXGG	UUCAAAU
Phenylalanine	GUCGGGA	GCUC	AGUUGGUA	GAGGA	CUGAAAA	GUGUC	ACCAG	UUCAAAU
Proline	AGGGAUG	GCGC	AGCUUGGUA	UUUGU	UUUGGGU	AUGUC	ACGGG	UUCAAAU
Serine	GGAGAGA	GCX_1–2_	X_4_GX_3–4_A	X_2_GX_1–2_	XUXGXAX	X_4_GX_15–19_	GAGGG	UUCGAAU
Threonine	XGCCX_0–4_	XCUC	AGXGGUA	XCGCX	X_3_GUAA	X_2_GUC	AUCGG	UUCX_3_U
Tryptophan	GCGCUCU	GUUC	AGUUCGGUA	UGGGU	CUCCAAA	AUGUC	GUAGG	UUCAAAU
Tyrosine	GGGUCGA	CCCG	AGCGGUUAA	ACGGA	CUGUAAA	GGCA	GCUGG	UUCAAAU
Valine	AGGGAUA	ACUC	AGCGGUA	UCACC	UUGACGU	AAGUC	AUCAG	UUCGAGC

Note: The asterisk (*****) shows the absence of conserved nucleotide consensus sequence in the respective region of the chloroplast tRNAs.

**Table 4:** The distribution of types of tRNAs in the three major clusters of the phylogenetic tree.

Integrated Clades	Types of tRNAs
clade I	tRNA^Ser^, tRNA^Leu^, tRNA^Arg^, tRNA^Met^, tRNA^Ala^, tRNA^Gly^, tRNA^Asp^, tRNA^Lys^, tRNA^Val^, tRNA^Ile^, tRNA^Thr^, tRNA^Pro^, tRNA^Gln^, tRNA^Cys^, tRNA^Phe^
clade II	tRNA^Ile^, tRNA^Leu^, tRNA^Gln^, tRNA^Tyr^, tRNA^His^, tRNA^Asn^, tRNA^Phe^, tRNA^Glu^, tRNA^Trp^, tRNA^Arg^
clade III	tRNA^Thr^, tRNA^Ser^, tRNA^Val^, tRNA^Met^, tRNA^Ile^

**Table 5:** Genomic distribution of biallelic single nucleotide polymorphic loci in the eight chloroplast genomes of diploid *Gossypium* plants.

Genome Region	Length (bp)	Value	%
Total substitutions	162,231	2709	1.67
Coding regions	79,244	906	1.14
Non-synonymous	/	681.58	0.86
Synonymous	/	224.42	0.28
dN/dS	/	3.04	/
Intron	21,443	299	1.39
Intergenic spacer	51,920	1504	2.90

## Supplementary Materials

The following are available online at https://www.mdpi.com/article/10.3390/genes12060822/s1. Figure S1: Duplication and loss events of the chloroplast tRNAs. Blue: duplication events; gray: loss events; D: duplication node; cD: conditional duplication node. Analysis indicates that the chloroplast tRNAs underwent frequent duplication events during their evolution with subsequent diversification, Table S1: Information registration of the analyzed tRNA genes, Table S2: Distribution of anti-codons in cotton chloroplast genome, Table S3: Nucleotide composition in acceptor arm (AA), D-arm (DA), D-loop (DL), anti-codon arm (ACA), anti-codon loop (ACL), variable loop (VL), pseudouridine arm (ΨA), and pseudouridine loop (ΨL) of chloroplast tRNA, Table S4: Results of transition and transversion rates of chloroplast tRNAs, Table S5: Loss events of the chloroplast tRNAs.
