# Supplementary material for: Novel Structural Variation and Evolutionary Characteristics of Chloroplast tRNA in Gossypium Plants
Source: Genes (Basel). 2021 May 27;12(6):822. doi: 10.3390/genes12060822 (PMC8228828; doi:10.3390/genes12060822)
Supplement: Supplementary file 1 [file genes-12-00822-s001.zip › Table S1.pdf]

Table S1 Information registration of the analyzed tRNA genes

Species	Name	Minimum	Maximum	Length
G. arboretum	trnI-CAU tRNA	158,107	158,180	74
	trnL-CAA tRNA	150,084	150,164	81
	trnV-GAC tRNA	143,580	143,651	72
	trnI-GAU tRNA	140,539	141,569	77
	trnA-UGC tRNA	139,606	140,475	73
	trnR-ACG tRNA	135,702	135,775	74
	trnN-GUU tRNA	134,999	135,070	72
	trnL-UAG tRNA	129,617	129,696	80
	trnN-GUU tRNA	113,861	113,932	72
	trnR-ACG tRNA	113,156	113,229	74
	trnA-UGC tRNA	108,456	109,325	73
	trnI-GAU tRNA	107,362	108,392	77
	trnV-GAC tRNA	105,280	105,351	72
	trnL-CAA tRNA	98,767	98,847	81
	trnI-CAU tRNA	90,751	90,824	74
	trnP-UGG tRNA	70,580	70,653	74
	trnW-CCA tRNA	70,328	70,400	73
	trnM-CAU tRNA	55,499	55,570	72
	trnV-UAC tRNA	54,622	55,303	85
	trnF-GAA tRNA	51,228	51,300	73
	trnL-UAA tRNA	50,162	50,831	87
	trnT-UGU tRNA	48,757	48,829	73
	trnS-GGA tRNA	47,238	47,324	87
	trnfM-CAU tRNA	38,806	38,879	74
	trnG-GCC tRNA	38,583	38,653	71
	trnS-UGA tRNA	37,152	37,244	93
	trnT-GGU tRNA	32,974	33,045	72
	trnE-UUC tRNA	32,085	32,157	73
	trnY-GUA tRNA	31,942	32,025	84
	trnD-GUC tRNA	31,366	31,439	74
	trnC-GCA tRNA	28,868	28,948	81
	trnR-UCU tRNA	10,181	10,252	72
	trnG-GCC tRNA	9,147	9,975	60
	trnS-GCU tRNA	8,172	8,259	88
	trnQ-UUG tRNA	6,969	7,040	72
	trnK-UUU tRNA	1,805	4,410	72
	trnH-GUG tRNA	3	77	75
G. anomalum	trnI-CAU tRNA	157,384	157,457	74
	trnL-CAA tRNA	149,349	149,429	81

	trnV-GAC tRNA	142,858	142,929	72
	trnI-GAU tRNA	139,818	140,847	77
	trnA-UGC	138,885	139,754	73
	trnR-ACG tRNA	134,998	135,071	74
	trnN-GUU tRNA	134,300	134,371	72
	trnL-UAG tRNA	128,934	129,013	80
	trnN-GUU tRNA	113,240	113,311	72
	trnR-ACG tRNA	112,540	112,613	74
	trnA-UGC	107,857	108,726	73
	trnI-GAU tRNA	106,764	107,793	76
	trnV-GAC tRNA	104,682	104,753	72
	trnL-CAA tRNA	98,182	98,262	81
	trnI-CAU tRNA	90,154	90,227	74
	trnP-UGG tRNA	70,026	70,099	74
	trnW-CCA tRNA	69,776	69,848	73
	trnM-CAU tRNA	54,918	54,990	73
	trnV-UAC tRNA	54,048	54,723	76
	trnF-GAA tRNA	50,736	50,808	73
	trnL-UAA tRNA	49,670	50,331	87
	trnT-UGU tRNA	48,265	48,337	73
	trnS-GGA tRNA	46,783	46,869	87
	trnfM-CAU tRNA	38,389	38,462	74
	trnG-GCC tRNA	38,166	38,236	71
	trnS-UGA tRNA	36,834	36,926	93
	trnT-GGU tRNA	32,659	32,730	72
	trnE-UUC tRNA	32,004	32,076	73
	trnY-GUA tRNA	31,861	31,944	84
	trnD-GUC tRNA	31,290	31,363	74
	trnC-GCA tRNA	28,806	28,886	81
	trnR-UCU tRNA	10,186	10,257	72
	trnG-UCC tRNA	9,156	9,995	80
	trnS-GCU tRNA	8,187	8,274	88
	trnQ-UUG tRNA	6,980	7,051	72
	trnK-UUU tRNA	1,837	4,443	72
	trnH-GUG tRNA	23	97	75
G. robinsonii	trnI-CAU tRNA	157,725	157,798	74
	trnL-CAA tRNA	149,701	149,781	81
	trnV-GAC tRNA	143,238	143,309	72
	trnI-GAU tRNA	140,193	141,222	77
	trnA-UGC	139,260	140,129	73
	trnR-ACG tRNA	135,368	135,441	74
	trnN-GUU tRNA	134,665	134,736	72
	trnL-UAG tRNA	129,376	129,455	80

	trnN-GUU tRNA	113,600	113,671	72
	trnR-ACG tRNA	112,895	112,968	74
	trnA-UGC	108,207	109,076	73
	trnI-GAU tRNA	107,114	108,143	77
	trnV-GAC tRNA	105,027	105,098	72
	trnL-CAA tRNA	98,555	98,635	81
	trnI-CAU tRNA	90,538	90,611	74
	trnP-UGG tRNA	70,242	70,315	74
	trnW-CCA tRNA	69,976	70,048	73
	trnM-CAU tRNA	55,185	55,256	72
	trnV-UAC tRNA	54,308	54,989	75
	trnF-GAA tRNA	50,938	51,010	73
	trnL-UAA tRNA	49,880	50,539	89
	trnT-UGU tRNA	48,427	48,499	73
	trnS-GGA tRNA	46,934	47,020	87
	trnfM-CAU tRNA	38,563	38,636	74
	trnG-GCC tRNA	38,341	38,411	71
	trnS-UGA tRNA	36,973	37,065	93
	trnT-GGU tRNA	32,783	32,854	72
	trnE-UUC tRNA	31,898	31,970	73
	trnY-GUA tRNA	31,755	31,838	84
	trnD-GUC tRNA	31,179	31,252	74
	trnC-GCA tRNA	28,954	29,034	81
	trnR-UCU tRNA	10,304	10,375	72
	trnG-GCC tRNA	9,259	10,099	71
	trnS-GCU tRNA	8,301	8,388	88
	trnQ-UUG tRNA	7,083	7,154	72
	trnK-UUU tRNA	1,917	4,537	72
	trnH-GUG tRNA	122	196	75
G. klotzschianum	trnI-CAU tRNA	157,975	158,048	74
	trnL-CAA tRNA	149,957	150,037	81
	trnV-GAC tRNA	143,457	143,528	72
	trnI-GAU tRNA	140,417	141,446	77
	trnA-UGC	139,484	140,353	73
	trnR-ACG tRNA	135,587	135,660	74
	trnN-GUU tRNA	134,889	134,960	72
	trnL-UAG tRNA	129,555	129,634	80
	trnN-GUU tRNA	113,792	113,863	72
	trnR-ACG tRNA	113,092	113,165	74
	trnA-UGC	108,399	109,268	73
	trnI-GAU tRNA	107,306	108,335	77
	trnV-GAC tRNA	105,224	105,295	72
	trnL-CAA tRNA	98,715	98,795	81

	trnI-CAU tRNA	90,704	90,777	74
	trnP-UGG tRNA	70,508	70,581	74
	trnW-CCA tRNA	70,251	70,323	73
	trnM-CAU tRNA	55,396	55,467	72
	trnV-UAC tRNA	54,519	55,200	75
	trnF-GAA tRNA	51,132	51,204	73
	trnL-UAA tRNA	50,059	50,724	87
	trnT-UGU tRNA	48,640	48,712	73
	trnS-GGA tRNA	47,127	47,213	87
	trnfM-CAU tRNA	38,728	38,801	74
	tRNA-Gly	38,505	38,575	71
	trnS-UGA tRNA	37,101	37,193	93
	trnT-GGU tRNA	32,916	32,987	72
	trnE-UUC tRNA	32,030	32,102	73
	trnY-GUA tRNA	31,887	31,970	84
	trnD-GUC tRNA	31,310	31,383	74
	trnC-GCA tRNA	28,812	28,892	81
	trnR-UCU tRNA	10,146	10,217	72
	trnG-GCC tRNA	9,103	9,948	68
	trnS-GCU tRNA	8,125	8,212	88
	trnQ-UUG tRNA	6,917	6,988	72
	trnK-UUU tRNA	1,793	4,354	73
	trnH-GUG tRNA	1	74	74
G. somalense	trnI-CAU tRNA	157,415	157,488	74
	trnL-CAA tRNA	149,399	149,479	81
	trnV-GAC tRNA	142,920	142,991	72
	trnI-GAU tRNA	139,880	140,909	77
	trnA-UGC	138,947	139,816	73
	trnR-ACG tRNA	135,065	135,138	74
	trnN-GUU tRNA	134,367	134,438	72
	trnL-UAG tRNA	129,020	129,099	80
	trnN-GUU tRNA	113,252	113,323	72
	trnR-ACG tRNA	112,552	112,625	74
	trnA-UGC	107,874	108,743	73
	trnI-GAU tRNA	106,781	107,810	77
	trnV-GAC tRNA	104,699	104,770	72
	trnL-CAA tRNA	98,211	98,291	81
	trnI-CAU tRNA	90,202	90,275	74
	trnP-UGG tRNA	70,023	70,096	74
	trnW-CCA tRNA	69,766	69,838	73
	trnM-CAU tRNA	54,981	55,052	72
	trnV-UAC tRNA	54,104	54,785	75
	trnF-GAA tRNA	50,793	50,865	73

	trnL-UAA tRNA	49,761	50,393	89
	trnT-UGU tRNA	48,360	48,432	73
	trnS-GGA tRNA	46,866	46,952	87
	trnfM-CAU tRNA	38,486	38,559	74
	trnG-GCC tRNA	38,262	38,332	71
	trnS-UGA tRNA	36,958	37,050	93
	trnT-GGU tRNA	32,785	32,856	72
	trnE-UUC tRNA	31,914	31,986	73
	trnY-GUA tRNA	31,771	31,854	84
	trnD-GUC tRNA	31,199	31,272	74
	trnC-GCA tRNA	28,718	28,798	81
	trnR-UCU tRNA	10,120	10,191	72
	trnG-GCC tRNA	9,092	9,915	71
	trnS-GCU tRNA	8,143	8,230	88
	trnQ-UUG tRNA	6,935	7,006	72
	trnK-UUU tRNA	1,786	4,384	72
	trnH-GUG tRNA	6	80	75
G. longicalyx	trnI-CAU tRNA	158,119	158,192	74
	trnL-CAA tRNA	150,096	150,176	81
	trnV-GAC tRNA	143,595	143,666	72
	trnI-GAU tRNA	140,553	141,583	77
	trnA-UGC	139,620	140,489	73
	trnR-ACG tRNA	135,693	135,766	74
	trnN-GUU tRNA	134,990	135,061	72
	trnL-UAG tRNA	129,626	129,705	80
	trnN-GUU tRNA	113,849	113,919	71
	trnR-ACG tRNA	113,143	113,215	73
	trnA-UGC	108,420	109,289	73
	trnI-GAU tRNA	107,326	108,356	76
	trnV-GAC tRNA	105,243	105,314	72
	trnL-CAA tRNA	98,733	98,813	81
	trnI-CAU tRNA	90,717	90,790	74
	trnP-UGG tRNA	70,517	70,590	74
	trnW-CCA tRNA	70,261	70,333	73
	trnM-CAU tRNA	55,474	55,545	72
	trnV-UAC tRNA	54,597	55,278	76
	trnF-GAA tRNA	51,235	51,307	73
	trnL-UAA tRNA	50,170	50,838	77
	trnT-UGU tRNA	48,749	48,821	73
	trnS-GGA tRNA	47,246	47,332	87
	trnfM-CAU tRNA	38,827	38,900	74
	trnG-GCC tRNA	38,604	38,674	71
	trnS-UGA tRNA	37,232	37,324	93

	trnT-GGU tRNA	33,039	33,110	72
	trnE-UUC tRNA	32,117	32,189	73
	trnY-GUA tRNA	31,974	32,057	84
	trnD-GUA tRNA	31,398	31,471	74
	trnC-GCA tRNA	28,898	28,978	81
	trnR-UCU tRNA	10,198	10,269	72
	trnG-UCC tRNA	9,128	10,002	70
	trnS-GCU tRNA	8,158	8,245	88
	trnQ-UUG tRNA	6,949	7,020	72
	trnK-UUU tRNA	1,794	4,394	72
	trnH-GUG tRNA	1	74	74
G. hirsutum	trnI-CAU tRNA	158,157	158,230	74
	trnL-CAA tRNA	150,134	150,214	81
	trnV-GAC tRNA	143,641	143,712	72
	trnI-GAU tRNA	140,600	141,630	77
	trnA-UGC	139,667	140,536	73
	trnR-ACG tRNA	135,759	135,832	74
	trnN-GUU tRNA	135,061	135,132	72
	trnL-UAG tRNA	129,681	129,760	80
	trnN-GUU tRNA	113,924	113,995	72
	trnR-ACG tRNA	113,224	113,297	74
	trnA-UGC	108,520	109,389	73
	trnI-GAU tRNA	107,426	108,456	77
	trnV-GAC tRNA	105,344	105,415	72
	trnL-CAA tRNA	98,842	98,922	81
	trnI-CAU tRNA	90,826	90,899	74
	trnP-UGG tRNA	70,646	70,719	74
	trnW-CCA tRNA	70,394	70,466	73
	trnM-CAU tRNA	55,555	55,626	72
	trnV-UAC tRNA	54,678	55,359	75
	trnF-GAA tRNA	51,288	51,360	73
	trnL-UAA tRNA	50,230	50,891	89
	trnT-UGU tRNA	48,828	48,900	73
	trnS-GGA tRNA	47,306	47,392	87
	trnfM-CAU tRNA	38,878	38,951	74
	trnG-GCC tRNA	38,655	38,725	71
	trnS-UGA tRNA	37,283	37,375	93
	trnT-GGU tRNA	33,077	33,148	72
	trnE-UUC tRNA	32,159	32,231	73
	trnY-GUA tRNA	32,016	32,099	84
	trnD-GUC tRNA	31,445	31,518	74
	trnC-GCA tRNA	28,902	28,982	81
	trnR-UCU tRNA	10,222	10,293	72

	trnG-GCC tRNA	9,164	9,996	71
	trnS-GCU tRNA	8,180	8,267	88
	trnQ-UUG tRNA	6,977	7,048	72
	trnK-UUU tRNA	1,822	4,435	72
	trnH-GUG tRNA	14	88	75
G. barbadense	trnI-CAU tRNA	158,188	158,261	74
	trnL-CAA tRNA	150,165	150,245	81
	trnV-GAC tRNA	143,671	143,742	72
	trnI-GAU tRNA	140,630	141,660	77
	trnA-UGC	139,697	140,566	73
	trnR-ACG tRNA	135,793	135,866	74
	trnN-GUU tRNA	135,095	135,166	72
	trnL-UAG tRNA	129,716	129,795	80
	trnN-GUU tRNA	113,964	114,035	72
	trnR-ACG tRNA	113,264	113,337	74
	trnA-UGC	108,564	109,433	73
	trnI-GAU tRNA	107,470	108,500	77
	trnV-GAC tRNA	105,388	105,459	72
	trnL-CAA tRNA	98,885	98,965	81
	trnI-CAU tRNA	90,869	90,942	74
	trnP-UGG tRNA	70,697	70,770	74
	trnW-CCA tRNA	70,447	70,519	73
	trnM-CAU tRNA	55,630	55,701	72
	trnV-UAC tRNA	54,753	55,434	75
	trnF-GAA tRNA	51,357	51,429	73
	trnL-UAA tRNA	50,293	50,960	89
	trnT-UGU tRNA	48,869	48,941	73
	trnS-GGA tRNA	47,350	47,436	87
	trnfM-CAU tRNA	38,918	38,991	74
	trnG-GCC tRNA	38,694	38,764	71
	trnS-UGA tRNA	37,214	37,306	93
	trnT-GGU tRNA	33,022	33,093	72
	trnE-UUC tRNA	32,108	32,180	73
	trnY-GUA tRNA	31,965	32,048	84
	trnD-GUC tRNA	31,389	31,462	74
	trnC-GCA tRNA	28,896	28,976	81
	trnR-UCU tRNA	10,233	10,304	72
	trnG-GCC tRNA	9,175	10,007	71
	trnS-GCU tRNA	8,191	8,278	88
	trnQ-UUG tRNA	6,983	7,054	72
	trnK-UUU tRNA	1,829	4,436	72
	trnH-GUG tRNA	14	88	75
G. bickii	trnI-CAU tRNA	157,295	157,368	74

	trnL-CAA tRNA	149,272	149,352	81
	trnV-GAC tRNA	142,803	142,874	72
	trnI-GAU tRNA	139,763	140,792	77
	trnA-UGC	138,830	139,699	73
	trnR-ACG tRNA	134,938	135,011	74
	trnN-GUU tRNA	134,235	134,306	72
	trnL-UAG tRNA	128,975	129,054	80
	trnN-GUU tRNA	113,190	113,261	72
	trnR-ACG tRNA	112,485	112,558	74
	trnA-UGC	107,797	108,666	73
	trnI-GAU tRNA	106,704	107,733	76
	trnV-GAC tRNA	104,622	104,693	72
	tnrL-CAA tRNA	98,144	98,224	81
	trnI-CAU tRNA	90,128	90,201	74
	trnP-UGG tRNA	70,000	70,073	74
	trnW-CCA tRNA	69,734	69,806	73
	trnM-CAU tRNA	54,819	54,891	73
	trnV-UAC tRNA	53,945	54,624	76
	trnF-GAA tRNA	50,602	50,674	73
	trnL-UAA tRNA	49,547	50,202	87
	trnT-UGU tRNA	48,169	48,241	73
	trnS-GGA tRNA	46,708	46,794	87
	trnG-GCC tRNA	38,084	38,154	71
	trnS-UGA tRNA	36,776	36,868	93
	trnT-GGU tRNA	32,595	32,666	72
	trnE-UUC tRNA	31,679	31,751	73
	trnY-GUA tRNA	31,536	31,619	84
	trnD-GUC tRNA	30,959	31,032	74
	trnC-GCA tRNA	28,730	28,810	81
	trnR-UCU tRNA	10,111	10,182	72
	trnG-UCC tRNA	9,053	9,915	70
	trnS-GCU tRNA	8,092	8,179	88
	trnQ-UUG tRNA	6,873	6,944	72
	trnK-UUU tRNA	1,805	4,414	72
	trnH-GUG tRNA	1	71	71
G. populifolium	trnI-CAU tRNA	157,311	157,384	74
	trnL-CAA tRNA	149,288	149,368	81
	trnV-GAC tRNA	142,829	142,900	72
	trnI-GAU tRNA	139,789	140,818	77
	trnA-UGC	138,856	139,725	73
	trnR-ACG tRNA	134,964	135,037	74
	trnN-GUU tRNA	134,261	134,332	72
	trnL-UAG tRNA	129,097	129,176	80

	trnN-GUU tRNA	113,310	113,381	72
	trnR-ACG tRNA	112,605	112,678	74
	trnA-UGC	107,917	108,786	73
	trnI-GAU tRNA	106,824	107,853	77
	trnV-GAC tRNA	104,742	104,813	72
	trnL-CAA tRNA	98,274	98,354	81
	trnI-CAU tRNA	90,258	90,331	74
	trnP-UGG tRNA	69,975	70,048	74
	trnW-CCA tRNA	69,709	69,781	73
	trnM-CAU tRNA	54,841	54,913	73
	trnV-UAC tRNA	53,966	54,646	75
	trnF-GAA tRNA	50,672	50,744	73
	trnL-UAA tRNA	49,616	50,274	87
	trnT-UGU tRNA	48,155	48,227	73
	trnS-GGA tRNA	46,650	46,736	87
	trnfM-CAU tRNA	38,208	38,281	74
	trnG-UCC tRNA	37,985	38,055	71
	trnS-UGA tRNA	36,711	36,803	93
	trnT-GGU tRNA	32,528	32,599	72
	trnE-UUC tRNA	31,641	31,713	73
	trnY-GUA tRNA	31,498	31,581	84
	trnD-GUC tRNA	31,054	31,127	74
	trnC-GCA tRNA	28,813	28,893	81
	trnR-UCU tRNA	10,180	10,251	72
	trnG-GCC tRNA	9,134	9,984	70
	trnS-GCU tRNA	8,166	8,253	88
	trnQ-UUG tRNA	6,956	7,027	72
	trnK-UUU tRNA	1,804	4,413	72
	trnH-GUG tRNA	1	74	74
