# Supplementary material for: Novel Structural Variation and Evolutionary Characteristics of Chloroplast tRNA in Gossypium Plants
Source: Genes (Basel). 2021 May 27;12(6):822. doi: 10.3390/genes12060822 (PMC8228828; doi:10.3390/genes12060822)
Supplement: Supplementary file 1 [file genes-12-00822-s001.zip › Table S2.pdf]

Table S2. Distribution of anti-codons in cotton chloroplast genome.

tRNA iso-acceptors	A2[1]	B1[2]	C2[3]	D3-k[4]	E2[5]	F1[6]	AD1[7]	AD2[8]	G1[9]	K2[10]
Alanine AGC	0	0	0	0	0	0	0	0	0	0
Alanine GGC	0	0	0	0	0	0	0	0	0	0
Alanine CGC	0	0	0	0	0	0	0	0	0	0
Alanine UGC	2	2	2	2	2	2	2	2	2	2
Glycine ACC	0	0	0	0	0	0	0	0	0	0
Glycine GCC	2	1	2	2	2	1	2	2	1	2
Glycine CCC	0	0	0	0	0	0	0	0	0	0
Glycine UCC	0	1	0	0	0	1	0	0	1	0
Proline AGG	0	0	0	0	0	0	0	0	0	0
Proline GGG	0	0	0	0	0	0	0	0	0	0
Proline CGG	0	0	0	0	0	0	0	0	0	0
Proline UGG	1	1	1	1	1	1	1	1	1	1
Threonine AGU	0	0	0	0	0	0	0	0	0	0
Threonine GGU	1	1	1	1	1	1	1	1	1	1
Threonine CGU	0	0	0	0	0	0	0	0	0	0
Threonine UGU	1	1	1	1	1	1	1	1	1	1
Valine AAC	0	0	0	0	0	0	0	0	0	0
Valine GAC	2	2	2	2	2	2	2	2	2	2
Valine CAC	0	0	0	0	0	0	0	0	0	0
Valine UAC	1	1	1	1	1	1	1	1	1	1
Serine AGA	0	0	0	0	0	0	0	0	0	0
Serine GGA	1	1	1	1	1	1	1	1	1	1
Serine CGA	0	0	0	0	0	0	0	0	0	0
Serine UGA	1	1	1	1	1	1	1	1	1	1
Serine ACU	0	0	0	0	0	0	0	0	0	0
Serine GCU	1	1	1	1	1	1	1	1	1	1
Arginine ACG	2	2	2	2	2	2	2	2	2	2
Arginine GCG	0	0	0	0	0	0	0	0	0	0
Arginine CCG	0	0	0	0	0	0	0	0	0	0
Arginine UCG	0	0	0	0	0	0	0	0	0	0
Arginine CCU	0	0	0	0	0	0	0	0	0	0
Arginine UCU	1	1	1	1	1	1	1	1	1	1
Leucine AAG	0	0	0	0	0	0	0	0	0	0
Leucine GAG	0	0	0	0	0	0	0	0	0	0
Leucine CAG	0	0	0	0	0	0	0	0	0	0
Leucine UAG	1	1	1	1	1	1	1	1	1	1
Leucine CAA	2	2	2	2	2	2	2	2	2	2
Leucine UAA	1	1	1	1	1	1	1	1	1	1
Phenylalanine AAA	0	0	0	0	0	0	0	0	0	0
Phenylalanine GAA	1	1	1	1	1	1	1	1	1	1
Asparagine AUU	0	0	0	0	0	0	0	0	0	0
Asparagine GUU	2	2	2	2	2	2	2	2	2	2
Lysine CUU	0	0	0	0	0	0	0	0	0	0
Lysine UUU	1	1	1	1	1	1	1	1	1	1
Aspartate AUC	0	0	0	0	0	0	0	0	0	0
Aspartate GUC	1	1	1	1	1	1	1	1	1	1
Glutamate CUC	0	0	0	0	0	0	0	0	0	0
Glutamate UUC	1	1	1	1	1	1	1	1	1	1
Histidine AUG	0	0	0	0	0	0	0	0	0	0
Histidine GUG	1	1	1	1	1	1	1	1	1	1

Glutamine CUG	0	0	0	0	0	0	0	0	0	0
Glutamine UUG	1	1	1	1	1	1	1	1	1	1
Isoleucine AAU	0	0	0	0	0	0	0	0	0	0
Isoleucine GAU	2	2	2	2	2	2	2	2	2	2
Isoleucine CAU	2	2	2	2	2	2	2	2	2	2
Isoleucine UAU	0	0	0	0	0	0	0	0	0	0
Methionine CAU	2	2	2	2	2	2	2	2	1	2
Tyrosine AUA	0	0	0	0	0	0	0	0	0	0
Tyrosine GUA	1	1	1	1	1	1	1	1	1	1
Cysteine ACA	0	0	0	0	0	0	0	0	0	0
Cysteine GCA	1	1	1	1	1	1	1	1	1	1
Tryptophan CCA	1	1	1	1	1	1	1	1	1	1
Suppressor CUA/ UUA/ UCA	0	0	0	0	0	0	0	0	0	0
Selenocysteine UCA	0	0	0	0	0	0	0	0	0	0
Total of anticodon types	28	29	28	28	28	29	28	28	29	28

[1]*Gossypium arboretum*; [2]*Gossypium anomalum*; [3]*Gossypium robinsonii*; [4]*Gossypium klotzschianum*; [5]*Gossypium somalense*; [6]*Gossypium longicalyx*; [7]*Gossypium hirsutum*; [8]*Gossypium barbadense*; [9]*Gossypium bickii*; [10]*Gossypium populifolium.*
