# Supplementary material for: Novel Structural Variation and Evolutionary Characteristics of Chloroplast tRNA in Gossypium Plants
Source: Genes (Basel). 2021 May 27;12(6):822. doi: 10.3390/genes12060822 (PMC8228828; doi:10.3390/genes12060822)
Supplement: Supplementary file 1 [file genes-12-00822-s001.zip › Table S3.pdf]

Table S3

Nucleotide composition in acceptor arm (AA), D-arm (DA), D-loop (DL), anti-codon arm (ACA), anti-codon loop (ACL), variable loop (VL), pseudouridine arm (ΨA), and pseudouridine loop (ΨL) of chloroplast tRNA

	AA	DA	DL	ACA	ACL	VL	ΨA	ΨL
Alanine								
Gossypium arboreum _108456	7	4	8	5	7	5	5	7
*Gossypium anomalum*_107857	7	4	8	5	7	5	5	7
*Gossypium robinsonii*_108207	7	4	8	5	7	5	5	7
*Gossypium klotzschianum*_108399	7	4	8	5	7	5	5	7
*Gossypium somalense*_107874	7	4	8	5	7	5	5	7
*Gossypium longicalyx*_139620	7	4	8	5	7	5	5	7
*Gossypium bickii*_107797	7	4	8	5	7	5	5	7
*Gossypium hirsutum*_108520	7	4	8	5	7	5	5	7
*Gossypium barbadense*_108564	7	4	8	5	7	5	5	7
*Gossypium populifolium*_107917	7	4	8	5	7	5	5	7
Gossypium arboreum _139606	7	4	8	5	7	5	5	7
*Gossypium anomalum*_138885	7	4	8	5	7	5	5	7
*Gossypium robinsonii*_139260	7	4	8	5	7	5	5	7
*Gossypium klotzschianum*_139484	7	4	8	5	7	5	5	7
*Gossypium somalense*_138947	7	4	8	5	7	5	5	7
*Gossypium longicalyx*_108420	7	4	8	5	7	5	5	7
*Gossypium bickii*_138830	7	4	8	5	7	5	5	7
*Gossypium hirsutum*_139667	7	4	8	5	7	5	5	7
*Gossypium barbadense*_139697	7	4	8	5	7	5	5	7
*Gossypium populifolium*_138856	7	4	8	5	7	5	5	7
Arginine								
*Gossypium arboreum*_10181	7	4	8	5	7	4	5	7
*Gossypium arboreum*_135702	7	4	9	5	7	5	5	5
*Gossypium arboreum*_113156	7	4	9	5	7	5	5	5
*Gossypium anomalum*_10186	7	4	8	5	7	4	5	7
*Gossypium anomalum*_112540	7	4	9	5	7	5	5	5
*Gossypium anomalum*_134998	7	4	9	5	7	5	5	5
*Gossypium robinsonii*_10304	7	4	8	5	7	4	5	7
*Gossypium robinsonii*_112895	7	4	9	5	7	5	5	5
*Gossypium robinsonii*_135368	7	4	9	5	7	5	5	5
*Gossypium klotzschianum*_10146	7	4	8	5	7	4	5	7
*Gossypium klotzschianum*_113092	7	4	9	5	7	5	5	5
*Gossypium klotzschianum*_135587	7	4	9	5	7	5	5	5
*Gossypium somalense*_10120	7	4	8	5	7	4	5	7
*Gossypium somalense*_112552	7	4	9	5	7	5	5	5
*Gossypium somalense*_135065	7	4	9	5	7	5	5	5
*Gossypium longicalyx*_113143	7	4	8	5	7	4	5	7
*Gossypium longicalyx*_10198	7	4	9	5	7	5	5	5
*Gossypium longicalyx*_113143	7	4	9	5	7	5	5	5
*Gossypium bickii*_10111	7	4	8	5	7	4	5	7
*Gossypium bickii*_112485	7	4	9	5	7	5	5	5

*Gossypium bickii*_134938	7	4	9	5	7	5	5	5
*Gossypium hirsutum*_113224	7	4	8	5	7	4	5	7
*Gossypium hirsutum*_10222	7	4	9	5	7	5	5	5
*Gossypium hirsutum*_135759	7	4	9	5	7	5	5	5
*Gossypium barbadense*_113264	7	4	8	5	7	4	5	7
*Gossypium barbadense*_10233	7	4	9	5	7	5	5	5
*Gossypium barbadense*_135793	7	4	9	5	7	5	5	5
*Gossypium populifolium*_10180	7	4	8	5	7	4	5	7
*Gossypium populifolium*_112605	7	4	9	5	7	5	5	5
*Gossypium populifolium*_134964	7	4	9	5	7	5	5	5
Asparagine								
*Gossypium arboreum*_ 113861	7	4	7	5	7	5	5	7
*Gossypium arboreum*_134999	7	4	7	5	7	5	5	7
*Gossypium anomalum*_113240	7	4	7	5	7	5	5	7
*Gossypium anomalum*_134300	7	4	7	5	7	5	5	7
*Gossypium robinsonii*_113600	7	4	7	5	7	5	5	7
*Gossypium robinsonii*_134665	7	4	7	5	7	5	5	7
*Gossypium klotzschianum*_113792	7	4	7	5	7	5	5	7
*Gossypium klotzschianum*_134889	7	4	7	5	7	5	5	7
*Gossypium somalense*_113252	7	4	7	5	7	5	5	7
*Gossypium somalense*_134367	7	4	7	5	7	5	5	7
*Gossypium longicalyx*_113849	7	4	7	5	7	5	5	7
*Gossypium longicalyx*_134990	7	4	7	5	7	5	5	7
*Gossypium bickii*_113190	7	4	7	5	7	5	5	7
*Gossypium bickii*_134235	7	4	7	5	7	5	5	7
*Gossypium hirsutum*_113924	7	4	7	5	7	5	5	7
*Gossypium hirsutum*_135061	7	4	7	5	7	5	5	7
*Gossypium barbadense*_113964	7	4	7	5	7	5	5	7
*Gossypium barbadense*_135095	7	4	7	5	7	5	5	7
*Gossypium populifolium*_113310	7	4	7	5	7	5	5	7
*Gossypium populifolium*_134261	7	4	7	5	7	5	5	7
Aspartate								
*Gossypium arboreum*_31364	7	4	9	5	7	5	5	7
*Gossypium anomalum*_31288	7	4	9	5	7	5	5	7
*Gossypium robinsonii*_31177	7	4	9	5	7	5	5	7
*Gossypium klotzschianum*_31308	7	4	9	5	7	5	5	7
*Gossypium somalense*_31197	7	4	9	5	7	5	5	7
*Gossypium longicalyx*_31396	7	4	9	5	7	5	5	7
*Gossypium bickii*_30957	7	4	9	5	7	5	5	7
*Gossypium hirsutum*_31439	7	4	9	5	7	5	5	7
*Gossypium barbadense*_31387	7	4	9	5	7	5	5	7
*Gossypium populifolium*_31052	7	4	9	5	7	5	5	7
Cysteine								
*Gossypium longicalyx*_28898	7	3	9	5	7	5	5	7
*Gossypium arboreum*_28868	7	3	9	5	7	5	5	7
*Gossypium anomalum*_28806	7	3	9	5	7	5	5	7
*Gossypium klotzschianum*_28812	7	3	9	5	7	5	5	7
*Gossypium robinsonii*_28954	7	3	9	5	7	5	5	7
*Gossypium somalense*_28718	7	3	9	5	7	5	5	7

*Gossypium hirsutum*_28902	7	3	9	5	7	5	5	7
*Gossypium bickii*_28730	7	3	9	5	7	5	5	7
*Gossypium barbadense*_28896	7	3	9	5	7	5	5	7
*Gossypium populifolium*_28813		3	9	5	7	5	5	7
Glutamate								
*Gossypium arboreum*_32085	7	4	9	5	7	4	5	7
*Gossypium anomalum*_32004	7	4	9	5	7	4	5	7
*Gossypium robinsonii*_31898	7	4	9	5	7	4	5	7
*Gossypium klotzschianum*_32030	7	4	9	5	7	4	5	7
*Gossypium somalense*_31914	7	4	7	5	7	5	5	7
*Gossypium longicalyx*_32117	7	4	7	5	7	5	5	7
*Gossypium bickii*_31679	7	4	9	5	7	4	5	7
*Gossypium hirsutum*_32159	7	4	9	5	7	4	5	7
*Gossypium barbadense*_32108	7	4	7	5	7	5	5	7
*Gossypium populifolium*_31641	7	4	7	5	7	5	5	7
Glutamine								
*Gossypium arboreum*_6969	7	3	9	4	7	6	5	7
*Gossypium anomalum*_6980	7	3	9	4	7	6	5	7
*Gossypium robinsonii*_7083	7	3	9	4	7	6	5	7
*Gossypium klotzschianum*_6917	7	3	9	4	7	6	5	7
*Gossypium somalense*_6935	7	3	9	4	7	6	5	7
*Gossypium longicalyx*_6949	7	3	9	4	7	6	5	7
*Gossypium bickii*_6873	7	3	9	4	7	6	5	7
*Gossypium hirsutum*_6968	7	3	9	4	7	6	5	7
*Gossypium barbadense*_6983	7	3	9	4	7	6	5	7
*Gossypium populifolium*_6956	7	3	9	5	7	4	5	7
Glycine								
*Gossypium arboreum*_38583	7	2	11	5	7	4	5	7
*Gossypium arboreum*_9147	7	2	11	5	7	4	5	7
*Gossypium anomalum*_38166	7	2	11	5	7	4	5	7
*Gossypium anomalum*_9156	7	2	11	5	7	4	5	7
*Gossypium robinsonii*_38341	7	2	11	5	7	4	5	7
*Gossypium robinsonii*_9259	7	2	11	5	7	4	5	7
*Gossypium klotzschianum*_38505	7	2	11	5	7	4	5	7
*Gossypium klotzschianum*_9103	7	2	11	5	7	4	5	7
*Gossypium somalense*_38262	7	2	11	5	7	4	5	7
*Gossypium somalense*_9092	7	2	11	5	7	4	5	7
*Gossypium longicalyx*_9128	7	2	11	5	7	4	5	7
*Gossypium longicalyx*_38604	7	2	11	5	7	4	5	7
*Gossypium bickii*_38084	7	2	11	5	7	4	5	7
*Gossypium bickii*_9053	7	2	11	5	7	4	5	7
*Gossypium hirsutum*_38655	7	2	11	5	7	4	5	7
*Gossypium hirsutum*_9164	7	2	11	5	7	4	5	7
*Gossypium barbadense*_38694	7	2	11	5	7	4	5	7
*Gossypium barbadense*_9175	7	2	11	5	7	4	5	7
*Gossypium populifolium*_37985	7	2	11	5	7	4	5	7
*Gossypium populifolium*_9134	7	2	11	5	7	4	5	7
Histidine								
*Gossypium arboreum*_3	7	3	11	5	7	5	5	7

*Gossypium anomalum*_23	7	3	11	5	7	5	5	7
*Gossypium robinsonii*_122	7	3	11	5	7	5	5	7
*Gossypium klotzschianum*_1	7	3	11	5	7	5	5	7
*Gossypium somalense*_6	7	3	11	5	7	5	5	7
*Gossypium longicalyx*_1	7	3	11	5	7	5	5	7
*Gossypium bickii*_1	7	3	11	5	7	5	5	7
*Gossypium hirsutum*_ 14	7	3	11	5	7	5	5	7
*Gossypium barbadense*_14	7	3	11	5	7	5	5	7
*Gossypium populifolium*_1	7	3	11	5	7	5	5	7
Leucine								
*Gossypium arboreum*_50162	7	3	11	5	7	2	5	7
*Gossypium arboreum*_129617	7	3	10	5	7	4	5	7
*Gossypium arboreum*_98767	7	3	10	5	7	4	5	7
*Gossypium arboreum*_150084	7	3	11	5	9	3	5	7
*Gossypium anomalum*_128934	7	3	11	5	7	2	5	7
*Gossypium anomalum*_98182	7	3	10	5	7	4	5	7
*Gossypium anomalum*_49670	7	3	10	5	7	4	5	7
*Gossypium anomalum*_149349	7	3	11	5	9	3	5	7
*Gossypium robinsonii*_98555	7	3	11	5	7	2	5	7
*Gossypium robinsonii*_49880	7	3	10	5	7	4	5	7
*Gossypium robinsonii*_129376	7	3	10	5	7	4	5	7
*Gossypium robinsonii*_149701	7	3	11	5	9	3	5	7
*Gossypium klotzschianum*_149957	7	3	11	5	7	2	5	7
*Gossypium klotzschianum*_129555	7	3	10	5	7	4	5	7
*Gossypium klotzschianum*_50059	7	3	10	5	7	4	5	7
*Gossypium klotzschianum*_98715	7	3	11	5	9	3	5	7
*Gossypium somalense*_49761	7	3	11	5	7	2	5	7
*Gossypium somalense*_98211	7	3	10	5	7	4	5	7
*Gossypium somalense*_129020	7	3	10	5	7	4	5	7
*Gossypium somalense*_149399	7	3	11	5	9	3	5	7
*Gossypium longicalyx*_129626	7	3	11	5	7	2	5	7
*Gossypium longicalyx*_50170	7	3	10	5	7	4	5	7
*Gossypium longicalyx*_98733	7	3	10	5	7	4	5	7
*Gossypium longicalyx*_150096	7	3	11	5	9	3	5	7
*Gossypium bickii*_49547	7	3	11	5	7	2	5	7
*Gossypium bickii*_128975	7	3	10	5	7	4	5	7
*Gossypium bickii*_98144	7	3	10	5	7	4	5	7
*Gossypium bickii*_149272	7	3	11	5	9	3	5	7
*Gossypium hirsutum*_150134	7	3	11	5	7	2	5	7
*Gossypium hirsutum*_50230	7	3	10	5	7	4	5	7
*Gossypium hirsutum*_129681	7	3	10	5	7	4	5	7
*Gossypium hirsutum*_98842	7	3	11	5	9	3	5	7
*Gossypium barbadense*_98885	7	3	11	5	7	2	5	7
*Gossypium barbadense*_129716	7	3	10	5	7	4	5	7
*Gossypium barbadense*_50293	7	3	10	5	7	4	5	7
*Gossypium barbadense*_150165	7	3	11	5	9	3	5	7
*Gossypium populifolium*_129097	7	3	11	5	7	2	5	7
*Gossypium populifolium*_98274	7	3	10	5	7	4	5	7
*Gossypium populifolium*_49616	7	3	10	5	7	4	5	7

*Gossypium populifolium*_149288	7	3	11	5	9	3	5	7
Lysine								
*Gossypium arboreum*_1805	7	4	7	4	7	6	5	7
*Gossypium anomalum*_1837	7	4	7	4	7	6	5	7
*Gossypium robinsonii*_1917	7	4	7	4	7	6	5	7
*Gossypium klotzschianum*_1793	7	4	7	4	7	6	5	7
*Gossypium somalense*_1786	7	4	7	4	7	6	5	7
*Gossypium longicalyx*_1794	7	4	7	4	7	6	5	7
*Gossypium bickii*_1805	7	4	7	4	7	6	5	7
*Gossypium hirsutum*_1822	7	4	7	4	7	6	5	7
*Gossypium barbadense*_1829	7	4	7	4	7	6	5	7
*Gossypium populifolium*_1804	7	4	7	4	7	6	5	7
Methionine								
*Gossypium arboreum*_55499	6	4	9	5	7	5	5	7
*Gossypium arboreum*_38806	6	4	9	5	7	5	5	7
*Gossypium anomalum*_54918	7	4	8	5	7	5	5	7
*Gossypium anomalum*_38389	6	4	9	5	7	5	5	7
*Gossypium robinsonii*_55185	6	4	9	5	7	5	5	7
*Gossypium robinsonii*_38563	7	4	8	5	7	5	5	7
*Gossypium klotzschianum*_38728	7	4	8	5	7	5	5	7
*Gossypium klotzschianum*_55396	6	4	9	5	7	5	5	7
*Gossypium somalense*_38486	6	4	9	5	7	5	5	7
*Gossypium somalense*_54981	7	4	8	5	7	5	5	7
*Gossypium longicalyx*_55474	6	4	9	5	7	5	5	7
*Gossypium longicalyx*_38827	7	4	8	5	7	5	5	7
*Gossypium bickii*_54819	7	4	8	5	7	5	5	7
*Gossypium hirsutum*_55555	7	4	8	5	7	5	5	7
*Gossypium hirsutum*_38878	6	4	9	5	7	5	5	7
*Gossypium barbadense*_38918	7	4	8	5	7	5	5	7
*Gossypium barbadense*_55630	6	4	9	5	7	5	5	7
*Gossypium populifolium*_38208	6	4	9	5	7	5	5	7
*Gossypium populifolium*_54841	7	4	8	5	7	5	5	7
Phenylalanine								
*Gossypium arboreum*_51228	7	4	8	5	7	5	5	7
*Gossypium anomalum*_50736	7	4	8	5	7	5	5	7
*Gossypium robinsonii*_50938	7	4	8	5	7	5	5	7
*Gossypium klotzschianum*_51132	7	4	8	5	7	5	5	7
*Gossypium somalense*_50793	7	4	8	5	7	5	5	7
*Gossypium longicalyx*_51235	7	4	8	5	7	5	5	7
*Gossypium bickii*_50602	7	4	8	5	7	5	5	7
*Gossypium hirsutum*_51235	7	4	8	5	7	5	5	7
*Gossypium barbadense*_51357	7	4	8	5	7	5	5	7
*Gossypium populifolium*_50672	7	4	8	5	7	5	5	7
Proline								
*Gossypium arboreum*_70580	7	4	9	5	7	5	5	7
*Gossypium anomalum*_70026	7	4	9	5	7	5	5	7
*Gossypium robinsonii*_70242	7	4	9	5	7	5	5	7
*Gossypium klotzschianum*_70508	7	4	9	5	7	5	5	7
*Gossypium somalense*_70023	7	4	9	5	7	5	5	7

*Gossypium longicalyx*_70517	7	4	9	5	7	5	5	7
*Gossypium bickii*_70000	7	4	9	5	7	5	5	7
*Gossypium hirsutum*_70646	7	4	9	5	7	5	5	7
*Gossypium barbadense*_70697	7	4	9	5	7	5	5	7
*Gossypium populifolium*_69975	7	4	9	5	7	5	5	7
Serine								
*Gossypium arboreum*_47238	7	3	11	5	7	4	5	7
*Gossypium arboreum*_37152	7	4	9	5	7	8	5	7
*Gossypium arboreum*_8172	7	3	11	4	7	3	5	7
*Gossypium anomalum*_46783	7	3	11	5	7	4	5	7
*Gossypium anomalum*_36834	7	4	9	5	7	8	5	7
*Gossypium anomalum*_8187	7	3	11	4	7	3	5	7
*Gossypium robinsonii*_46934	7	3	11	5	7	4	5	7
*Gossypium robinsonii*_36973	7	4	9	5	7	8	5	7
*Gossypium robinsonii*_8301	7	3	11	4	7	3	5	7
*Gossypium klotzschianum*_47127	7	3	11	5	7	4	5	7
*Gossypium klotzschianum*_37101	7	4	9	5	7	8	5	7
*Gossypium klotzschianum*_8125	7	3	11	4	7	3	5	7
*Gossypium somalense*_46866	7	3	11	5	7	4	5	7
*Gossypium somalense*_36958	7	4	9	5	7	8	5	7
*Gossypium somalense*_8143	7	3	11	4	7	3	5	7
*Gossypium longicalyx*_47246	7	3	11	5	7	4	5	7
*Gossypium longicalyx*_37232	7	4	9	5	7	8	5	7
*Gossypium longicalyx*_8158	7	3	11	4	7	3	5	7
*Gossypium bickii*_46708	7	3	11	5	7	4	5	7
*Gossypium bickii*_36776	7	4	9	5	7	8	5	7
*Gossypium bickii*_8092	7	3	11	4	7	3	5	7
*Gossypium hirsutum*_47306	7	3	11	5	7	4	5	7
*Gossypium hirsutum*_37283	7	4	9	5	7	8	5	7
*Gossypium hirsutum*_8180	7	3	11	4	7	3	5	7
*Gossypium barbadense*_47350	7	3	11	5	7	4	5	7
*Gossypium barbadense*_37214	7	4	9	5	7	8	5	7
*Gossypium barbadense*_8191	7	3	11	4	7	3	5	7
*Gossypium populifolium*_46650	7	3	11	5	7	4	5	7
*Gossypium populifolium*_36711	7	4	9	5	7	8	5	7
*Gossypium populifolium*_8166	7	3	11	4	7	3	5	7
Threonine								
*Gossypium arboreum*_48757	7	4	8	5	7	5	5	7
*Gossypium arboreum*_32974	7	4	7	5	7	5	5	7
*Gossypium anomalum*_48265	7	4	8	5	7	5	5	7
*Gossypium anomalum*_32659	7	4	7	5	7	5	5	7
*Gossypium robinsonii*_48427	7	4	8	5	7	5	5	7
*Gossypium robinsonii*_32783	7	4	7	5	7	5	5	7
*Gossypium klotzschianum*_48640	7	4	8	5	7	5	5	7
*Gossypium klotzschianum*_32916	7	4	7	5	7	5	5	7
*Gossypium somalense*_48360	7	4	8	5	7	5	5	7
*Gossypium somalense*_32785	7	4	7	5	7	5	5	7
*Gossypium longicalyx*_48749	7	4	8	5	7	5	5	7
*Gossypium longicalyx*_33039	7	4	7	5	7	5	5	7

*Gossypium bickii*_48169	7	4	8	5	7	5	5	7
*Gossypium bickii*_32595	7	4	7	5	7	5	5	7
*Gossypium hirsutum*_48828	7	4	8	5	7	5	5	7
*Gossypium hirsutum*_33077	7	4	7	5	7	5	5	7
*Gossypium barbadense*_48869	7	4	8	5	7	5	5	7
*Gossypium barbadense*_33022	7	4	7	5	7	5	5	7
*Gossypium populifolium*_48155	7	4	8	5	7	5	5	7
*Gossypium populifolium*_32528	7	4	7	5	7	5	5	7
Isoleucine								
*Gossypium arboreum*_158107	7	3	10	5	7	5	5	7
*Gossypium arboreum*_140539	7	4	7	5	7	5	5	7
*Gossypium arboreum*_107362	7	4	7	5	7	5	5	7
*Gossypium arboreum*_90751	7	3	10	5	7	5	5	7
*Gossypium anomalum*_157384	7	3	10	5	7	5	5	7
*Gossypium anomalum*_139818	7	4	7	5	7	5	5	7
*Gossypium anomalum*_106764	7	4	7	5	7	5	5	7
*Gossypium anomalum*_90154	7	3	10	5	7	5	5	7
*Gossypium robinsonii*_157725	7	3	10	5	7	5	5	7
*Gossypium robinsonii*_140193	7	4	7	5	7	5	5	7
*Gossypium robinsonii*_107114	7	4	7	5	7	5	5	7
*Gossypium robinsonii*_90538	7	3	10	5	7	5	5	7
*Gossypium klotzschianum*_157975	7	3	10	5	7	5	5	7
*Gossypium klotzschianum*_140417	7	4	7	5	7	5	5	7
*Gossypium klotzschianum*_107306	7	4	7	5	7	5	5	7
*Gossypium klotzschianum*_90704	7	3	10	5	7	5	5	7
*Gossypium somalense*_157415	7	3	10	5	7	5	5	7
*Gossypium somalense*_139880	7	4	7	5	7	5	5	7
*Gossypium somalense*_106781	7	4	7	5	7	5	5	7
*Gossypium somalense*_90202	7	3	10	5	7	5	5	7
*Gossypium longicalyx*_158119	7	3	10	5	7	5	5	7
*Gossypium longicalyx*_140553	7	4	7	5	7	5	5	7
*Gossypium longicalyx*_107326	7	4	7	5	7	5	5	7
*Gossypium longicalyx*_90717	7	3	10	5	7	5	5	7
*Gossypium bickii*_157295	7	3	10	5	7	5	5	7
*Gossypium bickii*_139763	7	4	7	5	7	5	5	7
*Gossypium bickii*_106704	7	4	7	5	7	5	5	7
*Gossypium bickii*_90128	7	3	10	5	7	5	5	7
*Gossypium hirsutum*_158157	7	3	10	5	7	5	5	7
*Gossypium hirsutum*_140600	7	4	7	5	7	5	5	7
*Gossypium hirsutum*_107426	7	4	7	5	7	5	5	7
*Gossypium hirsutum*_90826	7	3	10	5	7	5	5	7
*Gossypium barbadense*_158188	7	3	10	5	7	5	5	7
*Gossypium barbadense*_140630	7	4	7	5	7	5	5	7
*Gossypium barbadense*_107470	7	4	7	5	7	5	5	7
*Gossypium barbadense*_90869	7	3	10	5	7	5	5	7
*Gossypium populifolium*_157311	7	3	10	5	7	5	5	7
*Gossypium populifolium*_139789	7	4	7	5	7	5	5	7
*Gossypium populifolium*_106824	7	4	7	5	7	5	5	7
*Gossypium populifolium*_90258	7	3	10	5	7	5	5	7

Tryptophan								
*Gossypium arboreum*_70328	7	4	9	5	7	5	5	7
*Gossypium anomalum*_69776	7	4	9	5	7	5	5	7
*Gossypium robinsonii*_69976	7	4	9	5	7	5	5	7
*Gossypium klotzschianum*_70251	7	4	9	5	7	5	5	7
*Gossypium somalense*_69766	7	4	9	5	7	5	5	7
*Gossypium longicalyx*_70261	7	4	9	5	7	5	5	7
*Gossypium bickii*_69734	7	4	9	5	7	5	5	7
*Gossypium hirsutum*_70394	7	4	9	5	7	5	5	7
*Gossypium barbadense*_70447	7	4	9	5	7	5	5	7
*Gossypium populifolium*_69709	7	4	9	5	7	5	5	7
Tyrosine								
*Gossypium arboreum*_31942	7	4	9	5	7	3	5	7
*Gossypium anomalum*_31861	7	4	9	5	7	3	5	7
*Gossypium robinsonii*_31755	7	4	9	5	7	3	5	7
*Gossypium klotzschianum*_31887	7	4	9	5	7	3	5	7
*Gossypium somalense*_31771	7	4	9	5	7	3	5	7
*Gossypium longicalyx*_31974	7	4	9	5	7	3	5	7
*Gossypium bickii*_31536	7	4	9	5	7	3	5	7
*Gossypium hirsutum*_32016	7	4	9	5	7	3	5	7
*Gossypium barbadense*_31965	7	4	9	5	7	3	5	7
*Gossypium populifolium*_31498	7	4	9	5	7	3	5	7
Valine								
*Gossypium arboreum*_143580	7	4	7	5	7	5	5	7
*Gossypium arboreum*_105280	7	4	7	4	7	6	5	7
*Gossypium arboreum*_54622	7	4	8	4	7	6	5	7
*Gossypium anomalum*_142858	7	4	7	5	7	5	5	7
*Gossypium anomalum*_104682	7	4	7	4	7	6	5	7
*Gossypium anomalum*_54048	7	4	8	4	7	6	5	7
*Gossypium robinsonii*_143238	7	4	7	5	7	5	5	7
*Gossypium robinsonii*_105027	7	4	7	4	7	6	5	7
*Gossypium robinsonii*_54308	7	4	8	4	7	6	5	7
*Gossypium klotzschianum*_143238	7	4	7	5	7	5	5	7
*Gossypium klotzschianum*_105224	7	4	7	4	7	6	5	7
*Gossypium klotzschianum*_54519	7	4	8	4	7	6	5	7
*Gossypium somalense*_142920	7	4	7	5	7	5	5	7
*Gossypium somalense*_104699	7	4	7	4	7	6	5	7
*Gossypium somalense*_54104	7	4	8	4	7	6	5	7
*Gossypium longicalyx*_143595	7	4	7	5	7	5	5	7
*Gossypium longicalyx*_105243	7	4	7	4	7	6	5	7
*Gossypium longicalyx*_54597	7	4	8	4	7	6	5	7
*Gossypium bickii*_142803	7	4	7	5	7	5	5	7
*Gossypium bickii*_142803	7	4	7	4	7	6	5	7
*Gossypium bickii*_53945	7	4	8	4	7	6	5	7
*Gossypium hirsutum*_143641	7	4	7	5	7	5	5	7
*Gossypium hirsutum*_105344	7	4	7	4	7	6	5	7
*Gossypium hirsutum*_54678	7	4	8	4	7	6	5	7
*Gossypium barbadense*_143671	7	4	7	5	7	5	5	7
*Gossypium barbadense*_105388	7	4	7	4	7	6	5	7

*Gossypium barbadense*_54753	7	4	8	4	7	6	5	7
*Gossypium populifolium*_142829	7	4	7	5	7	5	5	7
*Gossypium populifolium*_104742	7	4	7	4	7	6	5	7
*Gossypium populifolium*_53966	7	4	8	4	7	6	5	7
