# Supplementary material for: Novel Structural Variation and Evolutionary Characteristics of Chloroplast tRNA in Gossypium Plants
Source: Genes (Basel). 2021 May 27;12(6):822. doi: 10.3390/genes12060822 (PMC8228828; doi:10.3390/genes12060822)
Supplement: Supplementary file 1 [file genes-12-00822-s001.zip › Table S4.pdf]

Table S4

Results of transition and transversion rates of chloroplast tRNAs.

From\To	A	U	C	G	From\To	A	U	C	G
		Alanine					Lysine		
A	-	8.34	8.34	**8.33**	A	-	3.37	3.37	**18.27**
U	8.34	-	**8.33**	8.34	U	3.37	-	**18.27**	3.37
C	8.34	**8.33**	-	8.34	C	3.37	**18.27**	-	3.37
G	**8.33**	8.34	8.34	-	G	**18.27**	3.37	3.37	-
		Arginine					Methionine		
A	-	5.15	5.15	**14.71**	A	-	6.89	6.89	**11.22**
U	5.15	-	**14.71**	5.15	U	6.89	-	**11.22**	6.89
C	5.15	**14.71**	-	5.15	C	6.89	**11.22**	-	6.89
G	**14.71**	5.15	5.15	-	G	**11.22**	6.89	6.89	-
		Asparagine					Phenylalanine		
A	-	8.34	8.34	**8.33**	A	-	8.34	8.34	**8.33**
U	8.34	-	**8.33**	8.34	U	8.34	-	**8.33**	8.34
C	8.34	**8.33**	-	8.34	C	8.34	**8.33**	-	8.34
G	**8.33**	8.34	8.34	-	G	**8.33**	8.34	8.34	-
		Aspartate					Proline		
A	-	8.34	8.34	**8.33**	A	-	8.34	8.34	**8.33**
U	8.34	-	**8.33**	8.34	U	8.34	-	**8.33**	8.34
C	8.34	**8.33**	-	8.34	C	8.34	**8.33**	-	8.34
G	**8.33**	8.34	8.34	-	G	**8.33**	8.34	8.34	-
		Cysteine					Serine		
A	-	4.99	4.99	**15.02**	A	-	8.29	8.29	**8.42**
U	4.99	-	**15.02**	4.99	U	8.29	-	**8.42**	8.29
C	4.99	**15.02**	-	4.99	C	8.29	**8.42**	-	8.29
G	**15.02**	4.99	4.99	-	G	**8.42**	8.29	8.29	-
		Glutamine					Threonine		
A	-	9.77	9.77	**5.46**	A	-	9.02	9.02	**6.95**
U	9.77	-	**5.46**	9.77	U	9.02	-	**6.95**	9.02
C	9.77	**5.46**	-	9.77	C	9.02	**6.95**	-	9.02
G	**5.46**	9.77	9.77	-	G	**6.95**	9.02	9.02	-
		Glutamate					Tryptophan		
A	-	4.51	4.51	**15.97**	A	-	0.00	0.00	**25.00**
U	4.51	-	**15.97**	4.51	U	0.00	-	**25.00**	0.00
C	4.51	**15.97**	-	4.51	C	0.00	**25.00**	-	0.00
G	**15.97**	4.51	4.51	-	G	**25.00**	0.00	0.00	-
		Glycine					Tyrosine		
A	-	7.89	7.89	**9.22**	A	-	8.34	8.34	**8.33**
U	7.89	-	**9.22**	7.89	U	8.34	-	**8.33**	8.34
C	7.89	**9.22**	-	7.89	C	8.34	**8.33**	-	8.34
G	**9.22**	7.89	7.89	-	G	**8.33**	8.34	8.34	-
		Histidine					Valine		

A	-	12.50	12.50	**0.00**	A	-	8.34	8.34	**8.33**
U	12.50	-	**0.00**	12.50	U	8.34	-	**8.33**	8.34
C	12.50	**0.00**	-	12.50	C	8.34	**8.33**	-	8.34
G	**0.00**	12.50	12.50	-	G	**8.33**	8.34	8.34	-
Isoleucine					Overall				
A	-	8.34	8.34	**8.33**	A	-	6.59	6.59	**11.82**
U	8.34	-	**8.33**	8.34	U	6.59	-	**11.82**	6.59
C	8.34	**8.33**	-	8.34	C	6.59	**11.82**	-	6.59
G	**8.33**	8.34	8.34	-	G	**11.82**	6.59	6.59	-
Leucine									
A	-	8.13	8.13	**8.74**					
U	8.13	-	**8.74**	8.13					
C	8.13	**8.74**	-	8.13					
G	**8.74**	8.13	8.13	-					
