# Supplementary material for: Novel Structural Variation and Evolutionary Characteristics of Chloroplast tRNA in Gossypium Plants
Source: Genes (Basel). 2021 May 27;12(6):822. doi: 10.3390/genes12060822 (PMC8228828; doi:10.3390/genes12060822)
Supplement: Supplementary file 1 [file genes-12-00822-s001.zip › Table S5.pdf]

Table S5

Loss events of the chloroplast tRNAs

tRNA Gene ID	Species	tRNA	Anti-codon	Lost in Species
NC_018113	*G. robinsonii*	Ala	UGC	*G. anomalum*
139260	*G. robinsonii*	Ala	UGC	*G. anomalum*
NC_033394	*G. klotzschianum*	Ala	UGC	*G. anomalum,*
NC_018110	*G. somalense*	Ala	UGC	*G. anomalum, G. robinsonii, G. klotzschianum*
NC_023216	*G. longicalyx*	Ala	UGC	*G. somalense, G. anomalum, G. robinsonii, G. klotzschianum*
139620	*G. longicalyx*	Ala	UGC	
NC_023214	*G. bickii*	Ala	UGC	*G. longicalyx, G. somalense, G. anomalum, G. robinsonii, G. klotzschianum*
138830	*G. bickii*	Ala	UGC	
NC_033398	*G. populifolium*	Ala	UGC	*G. bickii, G. longicalyx G. somalense G. anomalum G. robinsonii G. klotzschianum*
138856	*G. populifolium*	Ala	UGC	*G. bickii, G. arboreum, G. hirsutum, G. barbadense*
NC_016712	*G. arboreum*	Cys	GCG	
HQ901196	*G. hirsutum*	Ala	UGC	*G. arboreum*
139691	*G. hirsutum*	Ala	UGC	
HQ901199	*G. barbadense*	Ala	UGC	
139725	*G. barbadense*	Ala	UGC	*G,arboreum, G. hirsutum, G. bickii, G. longicalyx, G. somalense, G. populifolium, G. klotzschianum*
NC_023213	*G. anomalum*	Ala	UGC	
NC_016712	*G. arboreum*	Val	GAC	
HQ901196	*G. hirsutum*	Val	GAC	*G. arboreum, G. hirsutum, G. barbadense*
HQ901199	*G. barbadense*	Val	GAC	
NC_023213	*G. anomalum*	Val	GAC	
NC_018113	*G. robinsonii*	Val	GAC	*G. arboreum, G. hirsutum, G. barbadense*
NC_033394	*G. klotzschianum*	Val	GAC	

NC_018110	*G. somalense*	Val	UAC	
NC_033398	*G. populifolium*	Val	UAC	
143580	*G. arboreum*	Val	GAC	
143665	*G. hirsutum*	Val	GAC	
143697	*G. barbadense*	Val	GAC	
142858	*G. anomalum*	Val	GAC	
143238	*G. robinsonii*	Val	GAC	
105224	*G. klotzschianum*	Val	GAC	*G. arboreum, G. anomalum, G. robinsonii, G. hirsutum, G. barbadense*
143457	*G. klotzschianum*	Val	GAC	
142920	*G. somalense*	Val	GAC	
NC_023216	*G. longicalyx*	Val	GAC	*G. arboreum, G. somalense, G. anomalum, G. robinsonii, G. hirsutum, G. klotzschianum, G. barbadense*
143595	*G. longicalyx*	Val	GAC	
142803	*G. bickii*	Val	GAC	*G. arboreum G. longicalyx G. somalense G. anomalum G. robinsonii*
142829	*G. populifolium*	Val	GAC	*G. hirsutum G. populifolium G. klotzschianum G. barbadense*
NC_023214	*G. bickii*	Val	GAC	
157311	*G. bickii*	Val	CAU	
54841	*G. populifolium*	Val	CAU	*G. arboreum, G. somalense, G. robinsonii, G. hirsutum, G. klotzschianum, G. barbadense*
90153	*G. anomalum*	Val	CAU	
90716	*G. longicalyx*	Val	CAU	
NC_018113	*G. robinsonii*	Ile	CAU	
NC_018110	*G. somalense*	Ile	CAU	
HQ901199	*G. barbadense*	Ile	CAU	*G. klotzschianum*
NC_018113	*G. hirsutum*	Ile	CAU	
NC_016712	*G. arboreum*	Ile	CAU	
143238	*G. robinsonii*	Val	GAC	*G. klotzschianum*
142920	*G. somalense*	Val	GAC	

143697	*G. barbadense*	Val	GAC	
143580	*G. arboreum*	Val	GAC	
143665	*G. hirsutum*	Val	GAC	
NC_018113	*G. robinsonii*	Met	CAU	
NC_018110	*G. somalense*	Met	CAU	
NC_033394	*G. klotzschianum*	Met	CAU	*G. bickii, G. longicalyx, G. anomalum, G. populifolium*
HQ901196	*G. hirsutum*	Met	CAU	
HQ901199	*G. barbadense*	Met	CAU	
NC_016712	*G. arboreum*	Met	CAU	
NC_016712	*G. arboreum*	Asn	GUU	
134998	*G. arboreum*	Asn	GUU	*G. arboreum*
HQ901196	*G. hirsutum*	Asn	GUU	
135084	*G. hirsutum*	Asn	GUU	
HQ901199	*G. barbadense*	Asn	GUU	*G. arboreum, G. hirsutum*
135122	*G. barbadense*	Asn	GUU	
NC_023213	*G. anomalum*	Asn	GUU	
134299	*G. anomalum*	Asn	GUU	*G. arboreum G. hirsutum G. barbadense*
NC_018113	*G. robinsonii*	Asn	GUU	
134664	*G. robinsonii*	Asn	GUU	*G. arboreum G. anomalum G. hirsutum G. barbadense*
NC_033394	*G. klotzschianum*	Asn	GUU	
134888	*G. klotzschianum*	Asn	GUU	*G. arboreum G. anomalum G. robinsonii G. hirsutum G. barbadense*
NC_018110	*G. somalense*	Asn	GUU	
NC_023216	*G. longicalyx*	Asn	GUU	*G. arboreum, G. longicalyx, G. somalense, G. anomalum,*
NC_023214	*G. bickii*	Asn	GUU	
138830	*G. bickii*	Asn	GUU	*G. robinsonii, G. hirsutum, G. klotzschianum, G. barbadense*
113251	*G. somalense*	Asn	GUU	*G. arboreum G. anomalum G. robinsonii*

134366	*G. somalense*	Asn	GUU	*G. hirsutum* *G. klotzschianum* *G. barbadense*
134989	*G. longicalyx*	Asn	GUU	*G. arboreum* *G. longicalyx* *G. somalense* *G. anomalum*
113189	*G. bickii*	Asn	GUU	*G. robinsonii* *G. hirsutum* *G. klotzschianum*
NC_033398	*G. populifolium*	Asn	GUU	*G. arboreum* *G. bickii* *G. somalense* *G. anomalum*
134989	*G. longicalyx*	Asn	GUU	*G. robinsonii* *G. hirsutum* *G. populifolium*
157311	*G. populifolium*	Asn	GUU	*G. arboreum* *G. bickii* *G. longicalyx* *G. somalense* *G. anomalum* *G. robinsonii* *G. hirsutum*
38805	*G. arboreum*	Ile	CAU	
55572	*G. hirsutum*	Ile	CAU	
55629	*G. barbadense*	Ile	CAU	*G. arboreum* *G. hirsutum* *G. barbadense*
NC_023213	*G. anomalum*	Ile	CAU	
54918	*G. anomalum*	Ile	CAU	
38562	*G. robinsonii*	Ile	CAU	*G. arboreum* *G. anomalum* *G. robinsonii*
NC_033394	*G. klotzschianum*	Ile	CAU	
90703	*G. klotzschianum*	Ile	CAU	*G. hirsutum* *G. klotzschianum* *G. barbadense*
NC_023214	*G. bickii*	Ile	CAU	
38305	*G. bickii*	Ile	CAU	*G. arboreum* *G. longicalyx* *G. somalense* *G. anomalum* *G. robinsonii* *G. hirsutum* *G. klotzschianum*
NC_033398	*G. populifolium*	Ile	CAU	
90257	*G. populifolium*	Ile	CAU	*G. arboreum* *G. bickii* *G. longicalyx* *G. somalense* *G. anomalum* *G. robinsonii* *G. hirsutum*
90153	*G. anomalum*	Ile	CAU	*G. bickii* *G. longicalyx* *G. anomalum*

NC_023216	*G. longicalyx*	Ile	CAU					
54819	*G. bickii*	Ile	CAU					
55498	*G. arboreum*	Ile	CAU					
90750	*G. arboreum*	Ile	CAU					
90841	*G. hirsutum*	Ile	CAU					
158182	*G. hirsutum*	Ile	CAU	*G. bickii*	*G. arboreum*	*G. longicalyx*	*G. anomalum*	
90868	*G. barbadense*	Ile	CAU					
158188	*G. barbadense*	Ile	CAU	*G. bickii*	*G. arboreum*	*G. longicalyx*	*G. anomalum*	*G. hirsutum*
157384	*G. anomalum*	Ile	CAU	*G. bickii*	*G. arboreum*	G. longicalyx	*G. hirsutum*	*G. barbadense*
55184	*G. robinsonii*	Ile	CAU	*G. bickii*	*G. arboreum*	*G. longicalyx*		
90537	*G. robinsonii*	Ile	CAU	*G. anomalum*	*G. hirsutum*	*G. barbadense*		
NC_033394	*G. klotzschianum*	Ile	CAU	*G. bickii*	*G. arboreum*	*G. longicalyx*		
55395	*G. klotzschianum*	Ile	CAU	*G. anomalum*	*G. robinsonii*	*G. hirsutum*	*G. barbadense*	
38485	*G. somalense*	Ile	CAU	*G. bickii*	*G. arboreum*	*G. longicalyx*		
54980	*G. somalense*	Ile	CAU	*G. robinsonii*	*G. hirsutum*	*G. barbadense*	*G. anomalum*	
38826	*G. longicalyx*	Ile	CAU	*G. robinsonii*<br>*G. bickii*	*G. hirsutum*<br>*G. arboreum*	*G. klotzschianum*<br>*G. somalense*	*G. klotzschianum*<br>*G. anomalum*	
90127	*G. bickii*	Ile	CAU	*G. arboreum*<br>*G. robinsonii*	*G. longicalyx*<br>*G. hirsutum*	G. somalense	*G. anomalum*	
38207	*G. populifolium*	Ile	CAU	*G. bickii*	*G. arboreum*	*G. longicalyx*	*G. somalense*	
54841	*G. populifolium*	Ile	CAU	*G. anomalum*	*G. robinsonii*	*G. hirsutum*		
NC_033398	*G. populifolium*	Arg	ACG					
112605	*G. populifolium*	Arg	ACG	*G. longicalyx*				
NC_023216	*G. longicalyx*	Arg	ACG					

NC_016712	*G. arboreum*	Arg	ACG						
113156	*G. arboreum*	Arg	ACG						
HQ901196	*G. hirsutum*	Arg	ACG						
113241	*G. hirsutum*	Arg	ACG	*G. arboreum*	*G. longicalyx*				
HQ901199	*G. barbadense*	Arg	ACG						
113264	*G. barbadense*	Arg	ACG	*G. arboreum*	*G. longicalyx*	*G. hirsutum*			
NC_023213	*G. anomalum*	Arg	ACG						
112540	*G. anomalum*	Arg	ACG	*G. arboreum*	*G. longicalyx*	*G. hirsutum*	*G. barbadense*		
NC_018113	*G. robinsonii*	Arg	ACG						
112895	*G. robinsonii*	Arg	ACG	*G. arboreum*	*G. longicalyx*	*G. anomalum*	*G. hirsutum*	*G. barbadense*	
NC_033394	*G. klotzschianum*	Arg	ACG						
113092	*G. klotzschianum*	Arg	ACG	*G. arboreum*	*G. longicalyx*	*G. anomalum*	*G. robinsonii*	*G. hirsutum*	*G. barbadense*
NC_018110	*G. somalense*	Arg	ACG	*G. arboreum*	*G. longicalyx*	*G. anomalum*			
112552	*G. somalense*	Arg	ACG	*G. robinsonii*	*G. hirsutum*	*G. klotzschianum*	*G. barbadense*		
135692	*G. longicalyx*	Arg	ACG	*G. arboreum*<br>*G. robinsonii*	*G. somalense*<br>*G. hirsutum*	*G. anomalum*<br>*G. klotzschianum*	*G. barbadense*		
NC_023214	*G. bickii*	Arg	ACG	*G. arboreum*	*G. longicalyx*	*G. somalense*	*G. anomalum*		
112485	*G. bickii*	Arg	ACG	*G. robinsonii*	*G. hirsutum*	*G. klotzschianum*	*G. barbadense*		
NC_016712	*G. arboreum*	Leu	CAA						
150083	*G. arboreum*	Leu	CAA	*G. arboreum*					
HQ901196	*G. hirsutum*	Leu	CAA						
150158	*G. hirsutum*	Leu	CAA						
HQ901199	*G. barbadense*	Leu	CAA						
150164	*G. barbadense*	Leu	CAA	*G. arboreum*	*G. hirsutum*				
NC_023213	*G. anomalum*	Leu	CAA						

149348	*G. anomalum*	Leu	CAA	*G. arboreum*	*G. hirsutum*	*G. barbadense*			
NC_018113	*G. robinsonii*	Leu	CAA						
149700	*G. robinsonii*	Leu	CAA	*G. arboreum*	*G. anomalum*	*G. hirsutum*	*G. barbadense*		
NC_033394	*G. klotzschianum*	Leu	CAA						
98714	*G. klotzschianum*	Leu	CAA	*G. arboreum*	*G. anomalum*	*G. robinsonii*	*G. hirsutum*	*G. barbadense*	
NC_018110	*G. somalense*	Leu	CAA						
98210	*G. somalense*	Leu	CAA	*G. arboreum*	*G. anomalum*	*G. robinsonii*	*G. hirsutum*	*G. klotzschianum*	*G. barbadense*
NC_023216	*G. longicalyx*	Leu	CAA	*G. arboreum*	*G. somalense*	*G. anomalum*			
50170	*G. longicalyx*	Leu	CAA	*G. robinsonii*	*G. hirsutum*	*G. klotzschianum*	*G. barbadense*		
NC_023214	*G. bickii*	Leu	CAA	*G. longicalyx*	*G. somalense*	*G. anomalum*	*G. arboreum*		
98143	*G. bickii*	Leu	CAA	*G. robinsonii*	*G. hirsutum*	*G. klotzschianum*	*G. barbadense*		
NC_033398	*G. populifolium*	Leu	CAA	*G. arboreum,*	*G. bickii,*	*G. longicalyx,*	*G. somalense*		
98273	*G. populifolium*	Leu	CAA	*G. anomalum,*	*G. robinsonii,*	*G. hirsutum,*	*G. klotzschianum,*	*G. barbadense*	
98857	*G. hirsutum*	Leu	UAA						
98884	*G. barbadense*	Leu	UAA	*G. arboreum,*	*G. bickii,*	*G. longicalyx,*	*G. anomalum,*	*G. populifolium,*	*G. klotzschianum*
NC_018110	*G. somalense*	Leu	UAA						
49880	*G. robinsonii*	Leu	UAA						
50162	*G. arboreum*	Leu	UAA						
50059	*G. klotzschianum*	Leu	UAA						
49616	*G. populifolium*	Leu	UAA	*G. somalense,*	*G. robinsonii,*	*G. hirsutum,*	*G. barbadense*		
50170	*G. longicalyx*	Leu	UAA						
49547	*G. bickii*	Leu	UAA						
49670	*G. anomalum*	Leu	UAA						
NC_016712	*G. arboreum*	Met	CAU						
HQ901196	*G. hirsutum*	Met	CAU	*G. bickii*					
HQ901199	*G. barbadense*	Met	CAU						

NC_023213	*G. anomalum*	Met	CAU
NC_018113	*G. robinsonii*	Met	CAU
NC_033394	*G. klotzschianum*	Met	CAU
NC_018110	*G. somalense*	Met	CAU
NC_033398	*G. populifolium*	Met	CAU
NC_023216	*G. longicalyx*	Met	CAU
