# Supplementary figures and images for: Novel Structural Variation and Evolutionary Characteristics of Chloroplast tRNA in Gossypium Plants
Source: Genes (Basel). 2021 May 27;12(6):822. doi: 10.3390/genes12060822 (PMC8228828; doi:10.3390/genes12060822)

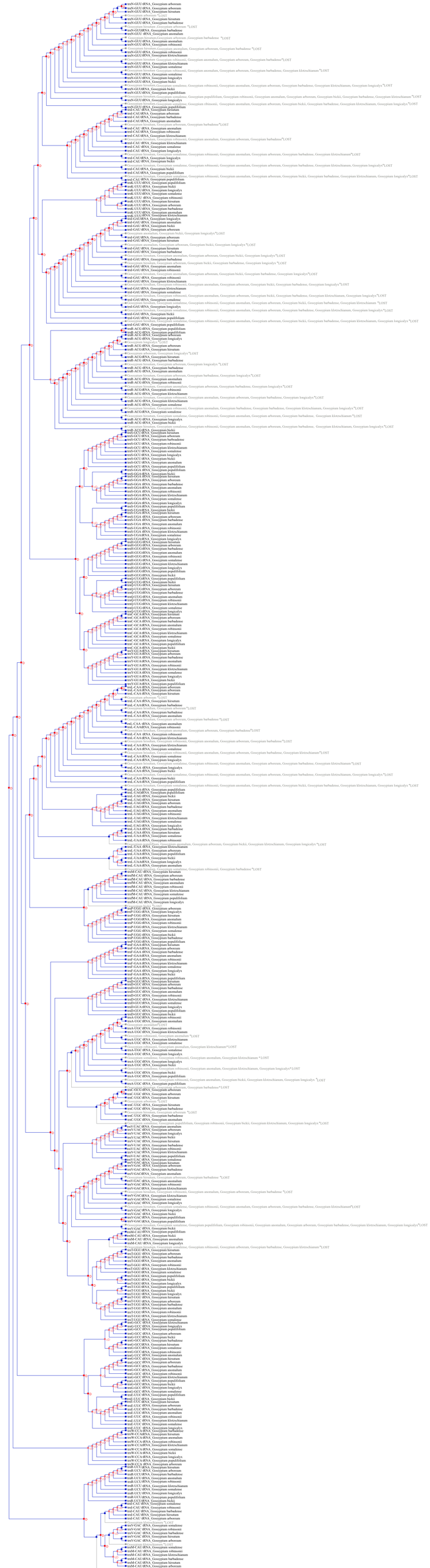

Supplement: Supplementary file 1 [file genes-12-00822-s001.zip › Figure S1.pdf]
